# Development of Conjugate Addition of Lithium Dialkylcuprates to Thiochromones: Synthesis of 2-Alkylthiochroman-4-ones and Additional Synthetic Applications

**DOI:** 10.3390/molecules23071728

**Published:** 2018-07-15

**Authors:** Shekinah A. Bass, Dynasty M. Parker, Tania J. Bellinger, Aireal S. Eaton, Angelica S. Dibble, Kaata L. Koroma, Sylvia A. Sekyi, David A. Pollard, Fenghai Guo

**Affiliations:** 1 Department of Chemistry, Winston Salem State University, 601 S. Martin Luther King Jr. Dr., Winston Salem, NC 27110, USA; sbass116@rams.wssu.edu (S.A.B.); dparker115@rams.wssu.edu (D.M.P.); tbellinger115@rams.wssu.edu (T.J.B.); aeaton114@rams.wssu.edu (A.S.E.); adibble114@rams.wssu.edu (A.S.D.); kkoroma116@rams.wssu.edu (K.L.K.); ssekyi116@rams.wssu.edu (S.A.S.); pollardda@wssu.edu (D.A.P.); 2 Biomedical Research Infrastructure Center, Winston Salem State University, Winston Salem, NC 27110, USA

**Keywords:** thiochroman-4-ones, conjugate addition, lithium dialkyl cuprates, thiochromones, 2-alkylthiochroman-4-ones

## Abstract

Lithium dialkylcuprates undergo conjugate addition to thiochromones to afford 2-alkylthiochroman-4-ones in good yields. This approach provide an efficient and general synthetic approach to privileged sulfur-containing structural motifs and valuable precursors for many pharmaceuticals, starting from common substrates-thiochromones. Good yields of 2-alkyl-substituted thiochroman-4-ones are attained with lithium dialkylcuprates, lithium alkylcyanocuprates or substoichiometric amount of copper salts. The use of commercially available inexpensive alkyllithium reagents will expedite the synthesis of a large library of 2-alkyl substituted thiochroman-4-ones for additional synthetic applications.

## 1. Introduction

**Scheme 1:**
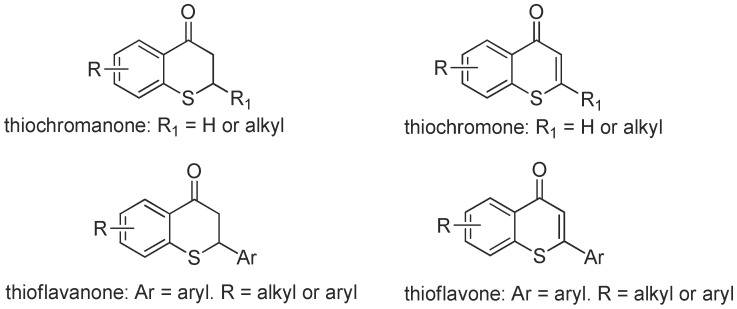
Structures of Thiochromanone, Thioflavone, Thiochromone, and Thioflavanones.

Sulfur-containing heterocycles are widely present in many bioactive natural products as well as pharmaceutical active molecules [1,2,3,4]. The sulfur-containing heterocycles are an understudied area when comparing to the oxygen-containing counterparts. In recent years, the development of efficient synthetic approaches to sulfur-containing compounds has gained much attention due to their widespread applications in biology, food chemistry, material science, and medicinal chemistry [1,2,3,4,5,6,7,8,9,10,11]. Sulfur-containing heterocycles, such as thiochromanone, thioflavanone, thiochromone, thioflavone, and their derivatives (Scheme 1) have been reported to display rich biological activities. For example, thioflavonoids, which are the sulfur analogues of flavonoids [12,13,14,15,16,17,18], display many biological activities, such as antimicrobial, antioxidant, inhibiting nitric oxide production, and antifungal et al. [3,19,20,21,22,23,24,25,26,27] Thiochroman-4-ones have been reported to display antifungal activities. Some thiochroman-4-one derivatives have been studied and shown to display the cytotoxic effect on tumor cells in vitro [28]. Recently, the in vitro antileishmanial and cytotoxic activities of some thiochroman-4-one derivatives have also been reported [29]. Many thiochromanone derivatives have been known to be effective “bioreductive alkylating agents”, inhibiting Ehrlich ascites tumor growth [21]. Other thiochroman-4-ones have shown the ability to kill tumor cells by inducing tumor cell apoptosis [30]. Thiochromanones, i.e., thiochroman-4-ones and 2-alkylthiochroman-4-ones, have become valuable synthons and precursors in organic synthesis in recent years. They are key precursors for certain bioactive antiproliferative agents [31]. Known as an important class of heterocycles [3,4], they are vital precursors of bioactive thiochroman-4-one 1,1-dioxanes, as well as benzothiazepins [20,21,32,33,34,35,36,37,38].

**Figure 1:**
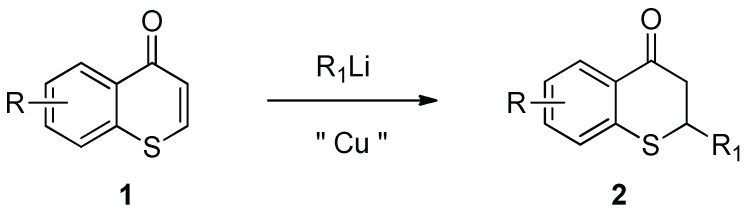
Conjugate Addition of Lithium Dialkylcuprates to Thiochromones.

Although some synthetic approaches to thiochroman-4-ones, thioflavone, and thiochromones have been reported in literature [28,39,40,41,42,43,44,45,46,47,48,49,50,51,52,53,54,55], research on efficient synthesis of 2-substituted thiochroman-4-ones is an underexplored area when compared to O-containing counterparts. Synthetic approaches to 2-substituted thiochroman-4-ones utilizing Friedel-Crafts acylation of thiopropanoic acid [56], hydrogenation of thiochromones [57,58,59], and intramolecular thio-Michael addition [60,61,62,63,64,65,66] have been reported. Recently, a rhodium-catalyzed alkyne hydroacylation/thio conjugate addition sequence in the synthesis of thiochroman-4-ones, including thioflavanones, has also been reported [67]. In another approach, Wang and coworkers reported an enantioselective Rh-catalyzed conjugate addition to thiochromones [68]. We also reported a rapid entry to thioflavanones via the conjugate addition of diarylcuprates to thiochromones recently [69]. While most of these approaches provided efficient approaches to thioflavanones (2-arylthiochroman-4-ones), they do not work particularly well in introducing the aliphatic groups to furnish the desired 2-alkylthiochroman-4-ones. For example, rhodium-catalyzed conjugate addition to thiochromones only works well with arylzinc reagents to introduce aryl groups to thiochromones and it is not compatible with alkylzinc reagents or aliphatic groups in general [68]. In an effort to develop a quick entry into 2-alkylthiochroman-4-ones by taking advantage of the readily available inexpensive alkyllithium reagents and copper salts, we now report the conjugate addition of lithium dialkylcuprates [70,71,72,73,74,75], prepared from the corresponding inexpensive commercially available alkyllithiums, to thiochromones **1** (see Supplementary Material for the preparation of these starting materials) to afford 2-alkylthiochroman-4-ones **2** in good yields (Figure 1).

## 2. Results and Discussions

We began our study with *n*-BuLi, copper (I) salt and thiochromone to investigate the reaction condition. No 1,4-adduct 2-*n*-butylthiochroman-4-one **4Aa** was formed with 0.3 equivalent of CuI or CuCN without any additive (Table 1, entries 1–2, 0%). Lithium cyanocuprate (i.e., *n*-BuCuCNLi) also fail to add to thiochromone with the recovery of unreacted thiochromone (Table 1, entry 3, 0%). Under similar reaction condition, more reactive Gilman reagents [76] (i.e., *n*-Bu_2_CuLi) afforded only a trace amount of 1,4-adduct **4Aa** (Table 1, entry 4). These results indicated that thiochromone is very sluggish towards the addition of lithium organocopper reagents without other additives/activators.

Lewis acids, such as trimethylsilyl chloride (TMSCl), have been known to accelerate 1,4-conjugate additions of both stoichiometric organocuprates and catalytic amount of copper (I) salts [77,78,79,80,81,82,83,84]. In our investigation, we found out that the yield of desired 1,4-adduct **4Aa** can be increased to 66% using 0.3 equivalent of CuI with the addition of TMSCl (Table 1, entry 5). Similar enhancement of reactivity was observed with 0.3 equivalent of CuCN in the presence of TMSCl (Table 1, entry 6). With the addition of TMSCl, lithium cyanocuprate reagent underwent smooth conjugate addition to thiochromone **3A** to afford 2-alkylthiochroman-4-one **4Aa** with 70% yield (Table 1, entry 7). Ultimately, lithium dialkylcuprate (i.e., *n*-Bu_2_CuLi) was found to be the most reactive and it afforded the highest yield of 1,4-adduct **4Aa** at 86% with the addition of TMSCl (Table 1, entry 8). The effect of other Lewis acid additives, such as TMSI and TMSOTf, were also investigated. Both TMSI and TMSOTf showed similar enhancement and promoted the conjugate addition of lithium di-*n*-butylcuprates to thiochroman-4-one with good yields (Table 1, entries 9–10, 85% and 82%).

**Scheme 2:**
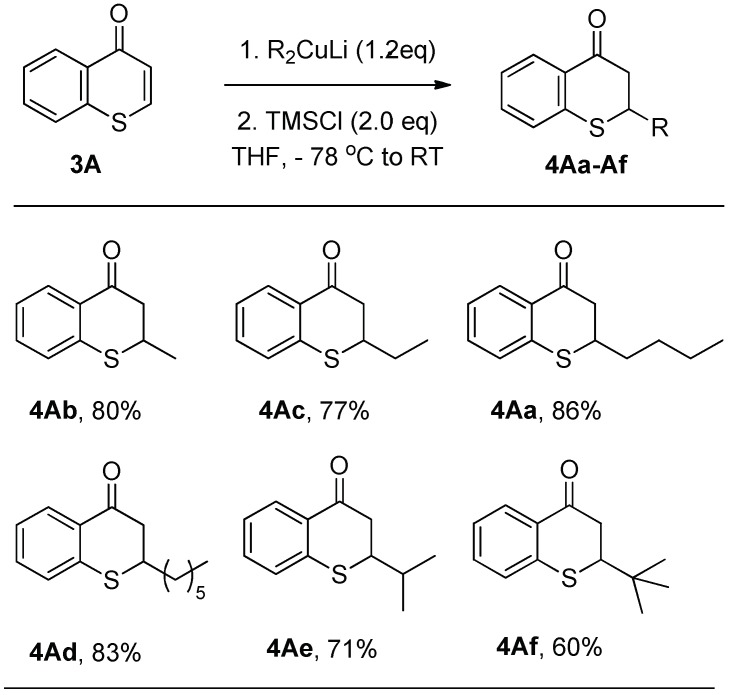
The Scope of Lithium Dialkylcuprates in Conjugate Addition to Thiochromone. a. All the reactions were performed using 1.2 equivalentof R_2_CuLi in the presence of 2.0 equivalent of TMSCl unless noted otherwise. b. RLi were commercially available. c. Yields are based on isolated products by flash column chromatography. d. Reactions were stirred for 12 h and warm up to room temperature before work up.

With the optimal reaction condition in hand, we examined the scope of lithium dialkylcuprates (i.e., R_2_CuLi) (Scheme 2, 60–86%). In general, a number of lithium dialkylcuprates underwent conjugate addition to thiochromone **3A** to afford 1,4-adducts **4Aa**-**Am** with good chemical yields (Scheme 2). Simple dialkylcuprates, such as dimethylcuprates, diethyl cuprate, di-*n*-butylcuprates, and di-*n*-hexylcuprates all add to **3A** smoothly to afford 1,4-adducts with good yields (Scheme 2, 81–86%). Lithium di-isopropylcuprate and di-*t*-buylcuprates also add to thiochromone **3A** with slightly lower yields (Scheme 2, 71% and 60%), indicating that organocuprates that are prepared from more hindered alkyllithium reagents are less reactive.

**Scheme 3:**
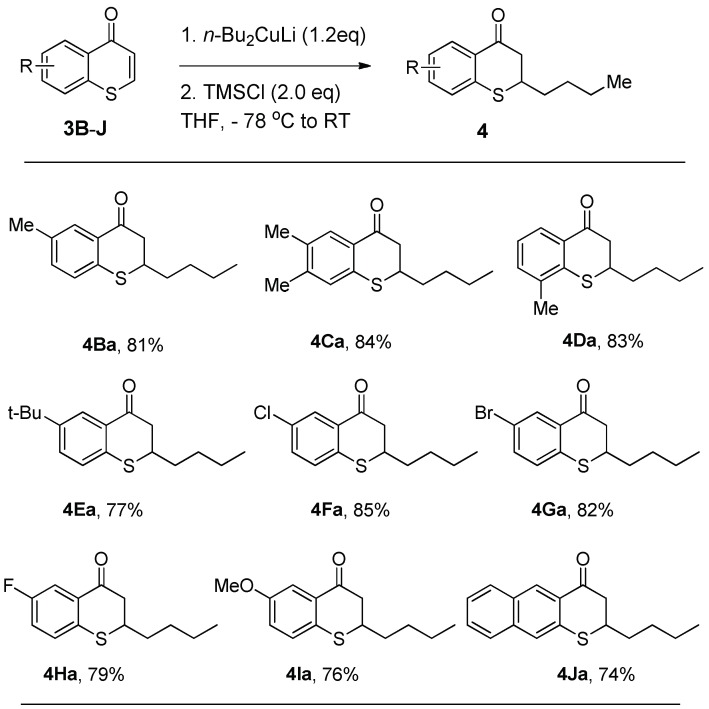
Reactions of Lithium di-*n*-butylcuprates with Substitututed Thiochromones. a. All the reactions were performed using 1.2 equivalentof n-Bu_2_CuLi in the presence of 2.0 equivalent of TMSCl unless noted otherwise. b. n-BuLi is commercially available. c. Yields are based on isolated products by flash column chromatography. d. Reactions were stirred for 12 h and warm up to room temperature before work up.

Having found the optimal reaction condition for conjugate addition to thiochromone **3A**, we next turned our attention to explore the scope of thiochromone substrates for the lithium dialkylcuprate conjugate addition. A number of substituted thiochromones **3B**-**J** were investigated. It was found that lithium di-*n*-butylcuprates (i.e., *n*-Bu_2_CuLi) readily add to substituted thiochromones **3B**-**J** to afford 1,4-adducts **4Ba**-**Ja** with 74–85% yields (Scheme 3). Thiochromones bearing simple substituents, such as methyl group, reacted with *n*-Bu_2_CuLi to afford **4Ba**-**Da** in 81–84% yields (Scheme 3). Bulky *t*-butyl group is also tolerated to afford 1,4-adduct **4Ea** with good yield. Thiochromones with halides F, Br, and Cl also work well with lithium di-*n*-butylcuprates (Scheme 3, 79–85%). Thiochromones with electron-donating groups, such as MeO-, also work well to afford 1,4-adduct **4Ia** in 76% yield (Scheme 3). Thiochromane **3J** with extended aromatic structure also undergo conjugate addition with *n*-Bu_2_CuLi to afford 1,4-adduct **4Ja** in 74% yield.

**Scheme 4:**
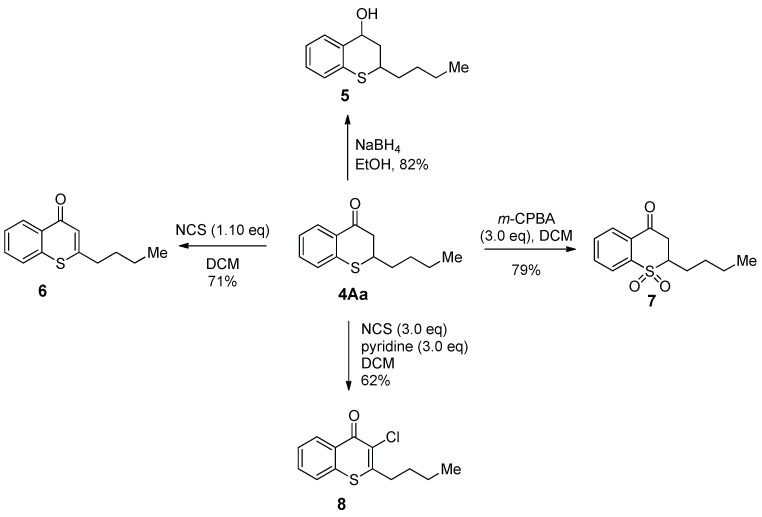
Synthetic Applications of 2-*n*-butyl Thiochroman-4-one.

Synthetic applications of 1,4-adducts: The 1,4-adducts-2-alkylthiochroman-4-ones can be utilized for additional synthetic applications (Scheme 4). For example, 2-*n*-butylthiochroman-4-one can be reduced to corresponding alcohol **5** by treatment with sodium borohydride in ethanol. Upon treatment with *N*-chlorosuccinimide (NCS) in dichloromethane, thiochroman-4-one **4Aa** was successfully converted into thiochromone **6** in 71% yield. 2-*n*-Butylthiochroman-4-one **4Aa** can be oxidized to sulfone **7** with an excess of *m*-chloroperbenzoic acid (*m*-CPBA) in dichloromethane (Scheme 4, 79%). It can also be functionalized to chlorinated thiochromone **8** upon treatment with excess of *N*-chlorosuccinimide (NCS, 3.0 equivalent) and pyridine (3.0 equivalent) (Scheme 4, 62%).

## 3. Materials and Methods

### 3.1. General Methods

The ^1^H, ^13^C, and ^19^F-NMR spectra were recorded on a BRUKER Ascend^TM^ 400 NMR spectrometer (Billerica, MA, USA), operating at 400 MHz for ^1^H and 100 MHz for ^13^C and 376 MHz for ^19^F. Samples for NMR spectra were dissolved in deuterated chloroform (with TMS). Analytical thin layer chromatography (TLC) was performed on silica gel plates, 60 µ mesh with F_254_ indicator. Visualization was accomplished by UV light (254 nm), and/or a 10% ethanol solution of phosphomolybdic acid and/or KMnO_4_ stain that is prepared by dissolving 1.5 g KMnO_4_, 10 g potassium carbonate, and 1.25 mL 10% sodium hydroxide in 200 mL water. Flash chromatography was performed with 230–400 µ silica gel. Infrared (IR) spectra were recorded on a Nicolet iS10 FT-IR spectrometer as neat samples (thin films).

### 3.2. Materials

Solvents and chemicals were obtained from commercial sources and used without further purification unless stated otherwise. Anhydrous tetrahydrofuran (THF) was purchased from Sigma Aldrich (Milwaukee, WI, USA). TMSCl was distilled from CaH_2_ under a positive N_2_ atmosphere. Alkyllithium reagents were purchased from Sigma Aldrich. All of the glassware was flamed-dried under high vacuum, purged with argon, and then cooled under a dry nitrogen atmosphere. Low temperature baths were prepared using dry ice-isopropanol slush bath mixtures. All organocuprate 1,4-conjugate addition reactions were conducted under a positive, dry argon atmosphere in anhydrous solvents in flasks that were fitted with a rubber septum.

### 3.3. General Procedure A: Conjugate Addition Reactions of Lithium Alkylcyanocuprates (RCuCNLi) with Thiochromones

To a flame-dried LiCl (51 mg, 1.2 mmol, 2.4 equivalent) under argon was added CuCN (53 mg, 0.6 mmol, 1.2 equivalent) and THF (1.5 mL). The resultant mixture was stirred for 10 mins at room temperature and then cooled to a −78 °C, followed by addition of alkyl lithium (0.6 mmol, 1.2 equivalent). The resultant solution was stirred for additional 30 mins at −78 °C under argon, followed by addition of thiochromone (0.5 mmol mixed with TMSCl (1.0 mmol) in THF (1.0 mL)) at −78 °C. The reaction mixture was allowed to warm up to room temperature during overnight stirring. Then, the reaction mixture was quenched with saturated aqueous NH_4_Cl (ca. 10.0 mL) and extracted with ethyl acetate (3 × 10.0 mL). The combined organic phase was washed with brine (ca. 15.0 mL), dried over anhydrous Na_2_SO_4_, filtered, concentrated in vacuo, and purified by flash column chromatography (silica gel, 0–2% ethyl acetate in hexane, *v*/*v*) to give pure compounds.

### 3.4. General Procedure B: Conjugate Addition Reactions of Lithium Dialkylcuprates (R_2_CuLi) with Thiochromones

General procedure B is identical to general procedure A except that double amount of alkyl lithium reagents (1.2 mmol, 2.4 equivalent) were used.

### 3.5. General Procedure C: Conjugate Addition Reactions of Alkyl Lithium Reagents with Thiochromone in the Presence of Substoichiometric Amount of CuI

To a CuI (0.30 equivalent) in THF (1.0 mL) under argon at 0 °C, was added alkyl lithium (1.2 equivalent). The resultant mixture was stirred for 30 min at room temperature 0 °C and then cooled to a −78 °C, followed by addition of thiochromone [0.5 mmol mixed with TMSCl (1.0 mmol) in THF (1.0 mL)]. The reaction mixture was allowed to warm up to room temperature during overnight stirring. Then, the reaction mixture was quenched with saturated aqueous NH_4_Cl (ca. 10.0 mL) and extracted with ethyl acetate (3 × 8.0 mL). The combined organic phase was washed with brine (ca. 10.0 mL), dried over anhydrous Na_2_SO_4_, filtered, concentrated in vacuo, and purified by flash column chromatography (silica gel, 0–2% ethyl acetate in hexane, *v*/*v*) to give pure compounds.

HRMS data for compounds **4Aa**, **4Ba**–**4Ja**, **5**, **7**, and **8** were analyzed by TOF MS. Compounds **4Ab**–**Ac**, **4Ad**–**Af**, and **6** have been fully characterized and reported [39,43,48,67].

#### 3.5.1. Synthesis of 2-*n*-Butylthiochroman-4-one (**4Aa**)

Employing General Procedure B, using *n*-BuLi (2.8 M, 0.43 mL, 1.2 mmol) and thiochromone (81 mg, 0.5 mmol), after purification by flash column chromatography (silica gel, 0–2% ethyl acetate:hexanes, *v*/*v*) gave light yellow oil **4Aa** (95 mg, 86%).

Alternatively, 2-*n*-butylthiochroman-4-one (**4Aa**) was prepared by employing General Procedure A, using *n*-BuLi (2.8 M, 0.22 mL, 0.6 mmol) and thiochromone (81 mg, 0.5 mmol), after purification by flash column chromatography (silica, 0–2% ethyl acetate:hexanes, *v*/*v*) gave light yellow oil **4Aa** (77 mg, 70%);

2-*n*-butylthiochroman-4-one (**4Aa**) was also prepared by employing General Procedure C, using *n*-BuLi (2.8 M, 0.22 mL, 0.6 mmol), CuI (29 mg) and thiochromone (81 mg, 0.5 mmol), after purification by flash column chromatography (silica gel, 0–2% ethyl acetate:hexanes, *v*/*v*) gave light yellow oil **4Aa** (72 mg, 66%): IR (neat) 3058 (w), 2955 (s), 2927 (s), 2857 (s), 1676 (s), 1588 (s), 1457 (m), 1435 (s), 1286 (s), 1231 (w), 1088 (m), 758 (m) cm^−1^; ^1^H-NMR (400 MHz, CDCl_3_) δ 0.94 (t, *J* = 7.2 Hz, 3H), 1.31–1.41 (m, 2H), 1.46 (quintet, *J* = 7.6 Hz, 2H), 1.74 (q, *J* = 7.6 Hz, 2H), 2.82 (dd, *J* = 11.2, 16.4 Hz, 1H), 3.07 (dd, *J* = 3.2, 16.4 Hz, 1H), 3.46–3.55 (m, 1H), 7.14–7.21 (m, 1H), 7.28 (d, *J* = 8.0 Hz, 1H), 7.37–7.43 (m, 1H), 7.14 (ddd, *J* = 0.4, 0.8, 8.0 Hz, 1H); ^13^C-NMR (100 MHz, CDCl_3_) δ 13.9, 22.3, 28.8, 34.2, 41.6, 46.3, 124.9, 127.6, 128.9, 130.7, 133.4, 141.7, 194.8; HRMS (EI-ion trap) *m*/*z*: [M]^+^ calcd. for C_13_H_16_OS, 220.0922; found 220.0918. 

#### 3.5.2. Synthesis of 6-Methyl-2-*n*-butylthiochroman-4-one (**4Ba**)

Employing General Procedure B and using 6-methylthiochromone (176 mg, 1.00 mmol) and *n*-BuLi (2.8 M, 0.86 mL, 2.40 mmol), after purification by flash column chromatography (silica gel, 0–2% ethyl acetate:hexanes, *v*/*v*) gave light yellow oil **4Ba** (190 mg, 81%); IR (neat) 3046 (w), 2955 (s), 2924 (s), 2856 (s), 1675 (s), 1602 (m), 1468 (m), 1398 (m), 1299 (w), 1278 (m), 1231 (w), 1097 (w), 814 (w) cm^−1^; ^1^H-NMR (400 MHz, CDCl_3_) δ 0.70 (t, *J* = 7.2 Hz, 3H), 1.03–1.14 (m, 2H), 1.17–1.30 (m, 2H), 1.50 (q, *J* = 7.6 Hz, 2H), 2.11 (s, 3H), 2.57 (dd, *J* = 11.2, 16.4 Hz, 1H), 2.82 (dd, *J* = 2.8, 16.4 Hz, 1H), 3.21–3.30 (m, 2H), 6.95 (d, *J* = 8.0 Hz, 1H), 7.00 (ddd, *J* = 0.4, 2.0, 8.4 Hz, 1H), 7.67–7.70 (m, 1H); ^13^C-NMR (100 MHz, CDCl_3_) δ 13.9, 20.8, 22.3, 28.9, 34.2, 41.7, 46.5, 127.6, 129.0, 130.5, 134.6, 134.7, 138.3, 195.1; HRMS (EI-ion trap) *m*/*z*: [M]^+^ calcd. for C_14_H_18_OS, 234.1078; found 234.1082.

#### 3.5.3. Synthesis of 6,7-Dimethyl-2-*n*-butylthiochroman-4-one (**4Ca**)

Employing General Procedure B and using 6,7-dimethylthiochromone (190 mg, 1.00 mmol) and and *n*-BuLi (2.8 M, 0.86 mL, 2.40 mmol), after purification by flash column chromatography (silica gel, 0–2% ethyl acetate:hexanes, *v*/*v*) gave light yellow solid **4Ca** (208 mg, 84%): m.p. 64.0–64.9 °C; IR (neat) 2956 (s) 2920 (s), 2860 (s), 1669 (s), 1599 (s), 1470 (m), 1447 (m), 1383 (m), 1370 (m), 1262 (s), 1147 (m), 1100 (m), 1023 (w), 864 (w) cm^−1^; ^1^H-NMR (400 MHz, CDCl_3_) δ 0.84 (t, *J* = 7.2 Hz, 3H), 1.21–1.31 (m, 2H), 1.36 (dt, *J* = 5.2, 7.6 Hz, 2H), 1.63 (q, *J* = 7.6 Hz, 2H), 2.16 (s, 3H), 2.17 (s, 3H), 2.68 (dd, *J* = 11.2, 16.4 Hz, 1H), 2.93 (dd, *J* = 3.2, 16.4 Hz, 1H), 3.30–3.43 (m, 1H), 7.14–7.21 (m, 1H), 7.28 (d, *J* = 8.0 Hz, 1H), 7.37–7.43 (m, 1H), 6.97 (s, 1H), 7.77 (s, 1H); ^13^C-NMR (100 MHz, CDCl_3_) δ 13.9, 19.2, 20.0, 22.3, 28.9, 34.2, 41.8, 46.4, 128.4, 128.6, 129.5, 133.8, 138.6, 143.7, 194.8; HRMS (EI-ion trap) *m*/*z*: [M]^+^ calcd. for C_15_H_20_OS, 248.1235; found 248.1236.

#### 3.5.4. Synthesis of 8-Methyl-2-*n*-butylthiochroman-4-one (**4Da**)

Employing General Procedure B and using 8-methylthiochromone (176 mg, 1.0 mmol) and *n*-BuLi (2.8 M, 0.43 mL, 1.2 mmol), after purification by flash column chromatography (silica gel, 0–2% ethyl acetate:hexanes, *v*/*v*) gave light yellow oil **4Da** (194 mg, 83%); IR (neat) 3061 (w), 2955 (m) 2926 (s), 2857 (m), 1676 (s), 1583 (m), 1449 (m), 1401 (m), 1379 (w), 1295 (m), 1279 (m), 1248 (w), 1056 (w), 1000 (w), 841 (w) 784 (w), 722 (w) cm^−1^; ^1^H-NMR (400 MHz, CDCl_3_) δ 0.73 (t, *J* = 7.2 Hz, 3H), 1.11–1.21 (m, 2H), 1.22–1.35 (m, 2H), 1.51–1.60 (m, 2H), 2.12 (s, 3H), 2.58 (dd, *J* = 11.6, 16.0 Hz, 1H), 2.83 (dd, *J* = 2.8, 16.0 Hz, 1H), 3.21–3.29 (m, 1H), 6.88 (t, *J* = 7.6 Hz, 1H), 7.09 (qd, *J* = 0.8, 8.0 Hz, 1H), 7.78 (qd, *J* = 0.4, 8.0 Hz, 1H); ^13^C-NMR (100 MHz, CDCl_3_) δ 13.9, 20.1, 22.4, 28.8, 34.4, 40.7, 45.7, 123.9, 126.6, 130.8, 134.5, 135.4, 141.3, 195.2; HRMS (EI-ion trap) *m*/*z*: [M]^+^ calcd. for C_14_H_18_OS, 234.1078; found 234.1075.

#### 3.5.5. Synthesis of 6-(*tert*-Butyl)-2-*n*-butylthiochroman-4-one (**4Ea**)

Employing General Procedure B and using 6-*tert*-butylthiochromone (86 mg, 0.42 mmol) and and *n*-BuLi (2.8 M, 0.36 mL, 1.01 mmol), after purification by flash column chromatography (silica gel, 0–2% ethyl acetate:hexanes, *v*/*v*) gave light yellow oil **4Ea** (89 mg, 77%); IR (neat) 3054 (w), 2955 (s), 2928 (s), 2869 (m), 1678 (s), 1596 (m), 1479 (m), 1463 (m), 1463 (m), 1252 (s), 1119 (m), 824 (m) cm^−1^; ^1^H-NMR (400 MHz, CDCl_3_) δ 0.84 (t, *J* = 7.2 Hz, 3H), 1.24 (s, 9H), 1.21–1.30 (m, 2H), 1.32–1.46 (m, 2H), 1.64 (q, *J* = 7.6 Hz, 2H), 2.72 (dd, *J* = 11.2, 16.4 Hz, 1H), 2.97 (dd, *J* = 3.2, 16.4 Hz, 1H), 3.35–3.45 (m, 1H), 7.13 (d, *J* = 8.40 Hz, 1H), 7.37 (dd, *J* = 2.4, 8.4 Hz, 1H), 8.04 (d, *J* = 2.40 Hz, 1H); ^13^C-NMR (100 MHz, CDCl_3_) δ 13.9, 22.3, 28.9, 31.1, 34.3, 34.6, 41.6, 46.5, 125.4, 127.4, 130.2, 131.2, 138.5, 148.1, 195.2; HRMS (EI-ion trap) *m*/*z*: [M]^+^ calcd. for C_17_H_24_OS, 276.1548; found 276.1544.

#### 3.5.6. Synthesis of 6-Chloro-2-*n*-butylthiochroman-4-one (**4Fa**)

Employing General Procedure B, and using 6-chlorothiochromone (130 mg, 0.66 mmol) and *n*-BuLi (2.8 M, 0.57 mL, 1.58 mmol), after purification by flash column chromatography (silica gel, 0–2% ethyl acetate:hexanes, *v*/*v*) gave light yellow oil **4Ea** (143 mg, 85%); IR (neat) 3057 (w), 2955 (s), 2927 (s), 2857 (m), 1682 (s), 1582 (m), 1452 (s), 1391 (m), 1293 (w), 1253 (m), 1224 (w), 1157 (w), 1094 (m), 898 (w), 815 (w), 730 (w) cm^−1^; ^1^H-NMR (400 MHz, CDCl_3_) δ 0.94 (t, *J* = 7.2 Hz, 3H), 1.32–1.40 (m, 2H), 1.41–1.50 (m, 2H), 1.74 (q, *J* = 7.6 Hz, 2H), 2.81 (dd, *J* = 11.2, 16.4 Hz, 1H), 3.06 (dd, *J* = 3.2, 16.4 Hz, 1H), 3.45–3.54 (m, 1H), 7.23 (d, *J* = 8.4 Hz, 1H), 7.36 (dd, *J* = 2.4, 8.4 Hz, 1H), 8.06 (d, *J* = 2.4 Hz, 1H); ^13^C-NMR (100 MHz, CDCl_3_) δ 13.9, 22.3, 28.8, 34.1, 41.7, 45.9, 128.5, 129.1, 131.1, 131.6, 133.4, 140.1, 193.6; HRMS (EI-ion trap) *m*/*z*: [M]^+^ calcd. for C_13_H_15_OSCl, 254.0532; found 254.0534.

#### 3.5.7. Synthesis of 6-Bromo-2-*n*-butylthiochroman-4-one (**4Ga**)

Employing General Procedure B and using 6-bromothiochromone (130 mg, 0.54 mmol) and *n*-BuLi (2.8 M, 0.39 mL, 1.08 mmol), after purification by flash column chromatography (silica gel, 0–2% ethyl acetate:hexanes, *v*/*v*) gave light yellow oil **4Ga** (132 mg, 82%); IR (neat) 3054 (w), 2955 (m) 2926 (s), 2856 (m), 1679 (s), 1574 (m), 1474 (m), 1450 (m), 1386 (m), 1291 (w), 1254 (m), 1224 (w), 1091 (m), 1054 (w), 898 (w), 813 (w) cm^−1^; ^1^H-NMR (400 MHz, CDCl_3_) δ 0.84 (t, *J* = 7.2 Hz, 3H), 1.21–1.31 (m, 2H), 1.32–1.41 (m, 2H), 1.64 (q, *J* = 7.6 Hz, 2H), 2.71 (dd, *J* = 11.2, 16.4 Hz, 1H), 2.97 (dd, *J* = 2.8, 16.4 Hz, 1H), 3.36–3.45 (m, 1H), 7.07 (d, *J* = 8.4 Hz, 1H), 7.40 (dd, *J* = 2.0, 8.4 Hz, 1H), 8.12 (d, *J* = 2.4 Hz, 1H); ^13^C-NMR (100 MHz, CDCl_3_) δ 13.9, 22.3, 28.8, 34.1, 41.7, 45.8, 118.6, 129.3, 131.5, 131.9, 136.2, 140.7, 193.5; HRMS (EI-ion trap) *m*/*z*: [M]^+^ calcd. for C_13_H_15_OSBr, 298.0027; found 298.0033.

#### 3.5.8. Synthesis of 6-Fluoro-2-*n*-butylthiochroman-4-one (**4Ha**)

Employing General Procedure B and using 6-fluorothiochromone (100 mg, 0.56 mmol) and *n*-BuLi (2.8 M, 0.48 mL, 1.34 mmol), after purification by flash column chromatography (silica gel, 0–2% ethyl acetate:hexanes, *v*/*v*) gave light yellow oil **4Ha** (105 mg, 79%); IR (neat) 3066 (w), 2957 (m) 2928 (s), 2858 (m), 1682 (s), 1601 (m), 1464 (s), 1404 (s), 1303 (m), 1262 (s), 1223 (w), 1196 (w), 1089 (w), 895 (w), 817 (w) cm^−1^; ^1^H-NMR (400 MHz, CDCl_3_) δ 0.94 (t, *J* = 7.2 Hz, 3H), 1.32–1.41 (m, 2H), 1.42–1.50 (m, 2H), 1.74 (q, *J* = 7.6 Hz, 2H), 2.81 (dd, *J* = 11.2, 16.4 Hz, 1H), 3.07 (dd, *J* = 2.8, 16.4 Hz, 1H), 3.46–3.55 (m, 1H), 7.15 (ddd, *J* = 2.8, 8.0, 8.4 Hz, 1H), 7.27 (dd, *J* = 5.2, 8.8 Hz, 1H), 7.79 (dd, *J* = 3.2, 9.6 Hz, 1H); ^13^C-NMR (100 MHz, CDCl_3_) δ 13.9, 22.3, 28.8, 34.0, 41.8, 46.0, 114.9 (d, *J* = 22 Hz), 121.4 (d, *J* = 22 Hz), 129.4 (d, *J* = 7 Hz), 132.0 (d, *J* = 6 Hz), 136.9 (d, *J* = 3 Hz), 160.4 (d, *J* = 244 Hz), 193.8; ^19^F NMR (376 MHz, CDCl_3_) δ - 116.8 (quintet, *J* = 3.76 Hz); HRMS (EI-ion trap) *m*/*z*: [M]^+^ calcd. for C_13_H_15_OSF, 238.0828; found 238.0827.

#### 3.5.9. Synthesis of 6-Methoxy-2-*n*-butylthiochroman-4-one (**4Ia**)

Employing General Procedure B and using 6-methoxydimethylthiochromone (76 mg, 0.4 mmol) and and *n*-BuLi (2.8 M, 0.34 mL, 0.96 mmol), after purification by flash column chromatography (silica gel, 0–2% ethyl acetate:hexanes, *v*/*v*) gave light yellow oil **4Ia** (76 mg, 76%); IR (neat) 3065 (w), 2955 (m) 2925 (s), 2854 (m), 1675 (s), 1599 (m), 1471 (s), 1403 (m), 1323 (w), 1273 (s), 1222 (s), 1180 (w), 1099 (w), 1027 (m), 870 (w), 820 (w) cm^−1^; ^1^H-NMR (400 MHz, CDCl_3_) δ 0.84 (t, *J* = 7.2 Hz, 3H), 1.21–1.31 (m, 2H), 1.39 (td, *J* = 2.0, 14.8 Hz, 2H), 1.64 (q, *J* = 7.6 Hz, 2H), 2.71 (dd, *J* = 11.2, 16.4 Hz, 1H), 2.97 (dd, *J* = 2.8, 16.4 Hz, 1H), 3.30–3.49 (m, 1H), 3.75 (s, 3H), 6.94 (dd, *J* = 2.8, 8.8 Hz, 1H), 7.11 (dd, *J* = 0.4, 8.8 Hz, 1H), 7.52 (d, *J* = 2.8 Hz, 1H); ^13^C-NMR (100 MHz, CDCl_3_) δ 13.9, 22.3, 28.9, 34.1, 41.9, 46.5, 55.6, 111.0, 122.5, 129.0, 131.4, 133.1, 157.3, 194.8; HRMS (EI-ion trap) *m*/*z*: [M]^+^ calcd. for C_14_H_18_O_2_S, 250.1028; found 250.1029.

#### 3.5.10. Synthesis of 2-*n*-Butyl-2,3-dihydro-4*H*-benzo[g]thiochromen-4-one (**4Ja**)

Employing General Procedure B and using 6,7-dimethylthiochromone (106 mg, 0.5 mmol) and and *n*-BuLi (2.8 M, 0.43 mL, 1.2 mmol), after purification by flash column chromatography (silica gel, 0–2% ethyl acetate:hexanes, *v*/*v*) gave light yellow oily liquid **4Ja** (100 mg, 74%); IR (neat) 3051 (w), 2954 (s) 2926 (s), 2856 (m), 1660 (s), 1613 (m), 1590 (m), 1549 (w), 1504 (m), 1464 (w), 1422 (m), 1335 (m), 1215 (m), 1111 (m), 872 (w), 813 (m), 779 (w), 746 (m) cm^−1^; ^1^H-NMR (400 MHz, CDCl_3_) δ 0.86 (t, *J* = 7.2 Hz, 3H), 1.25–1.35 (m, 2H), 1.36–1.46 (m, 2H), 1.70 (q, *J* = 7.6 Hz, 2H), 2.87 (dd, *J* = 11.2, 15.2 Hz, 1H), 3.09 (dd, *J* = 3.6, 15.2 Hz, 1H), 3.45–3.54 (m, 1H), 7.19 (d, *J* = 8.4 Hz, 1H), 7.34–7.40 (m, 1H), 7.52 (ddd, *J* = 1.6, 6.8, 8.8 Hz, 1H), 7.64–7.68 (m, 1H), 7.71 (d, *J* = 8.8 Hz, 1H), 9.12 (dd, *J* = 0.8, 8.8 Hz, 1H); ^13^C-NMR (100 MHz, CDCl_3_) δ 13.9, 22.4, 28.8, 34.2, 41.3, 47.7, 125.1, 125.4, 125.6, 125.8, 128.4, 129.2, 131.7, 132.3, 133.7, 144.7, 197.0; HRMS (EI-ion trap) *m*/*z*: [M]^+^ calcd. for C_17_H_18_OS, 270.1078; found 270.1074.

### 3.6. Synthesis of 2-n-Buylthiochroman-4-ol *(**5**)*

To a dry ethanol solution (1.5 mL) of 2-*n*-butylthiochroman-4-one (0.5 mmol, 110 mg) under argon, sodium borohydride (0.25 mmol, 10 mg) was added portion-wise. The resultant mixture was stirred at room temperature for 2 h. Then solvent was evaporated, ice water (10 mL) was added, and the mixture was acidified with 10% HCl to pH = 1–2. It was then extracted with ethyl acetate (3 × 8 mL) and organic layers were combined, washed with brine (15 mL). The organic layer was dried (Na_2_SO_4_), filtered and evaporated under vacuum to give crude product. The crude product was then purified by flash column chromatography (silica gel, 10% ethyl acetate:hexanes, *v*/*v*) to give 2-*n*-butylthiochroman-4-ol **5** as white solid (91 mg, 82%): m.p. 64.1–65.2 °C; IR (neat) 3317 (br s), 3065 (w), 2958 (s), 2924 (s), 2855 (s), 1591 (w), 1566 (w), 1466 (m), 1433 (s), 1349 (w), 1308 (m), 1263 (m), 1196 (w), 1059 (m), 1034 (m), 1016 (m), 978 (w), 759 (m), 750 (s), 730 (m), 688 (w) cm^−1^; ^1^H-NMR (400 MHz, CDCl_3_) δ 0.96 (t, *J* = 7.2 Hz, 3H), 1.36–1.50 (m, 3H), 1.60–1.75 (m, 2H), 1.75–1.86 (m, 1H), 2.27 (d, *J* = 8 Hz, 1H), 2.46 (ddd, *J* = 3.2, 4.4, 12.8 Hz, 1H), 3.38–3.48 (m, 1H), 7.07–7.16 (m, 3H), 7.53–7.59 (m, 1H); ^13^C-NMR (100 MHz, CDCl_3_) δ 14.0, 22.5, 28.9, 36.3, 40.0, 40.3, 69.2, 124.4, 126.1, 126.2, 127.6, 133.3, 137.1; HRMS (EI-ion trap) *m*/*z*: [M]^+^ calcd. for C_13_H_18_OS, 222.1078; found 222.1084.

### 3.7. Synthesis of 2-n-Butyl-thiochromen-4-one 1,1-dioxide *(**7**)*

To a dry DCM (dichloromethane) solution of 2-*n*-butylthiochroman-4-one (0.5 mmol) under Ar atmosphere in a 50 mL RB (round-bottom) flask, was added excess 3-meta-chloroperoxybenzoic acid (*m*-CPBA, 3.0 equivalent, 1.5 mmol, 259 mg). The resultant mixture was stirred at room temperature until the reaction is complete by TLC monitoring (5 h). Then the reaction mixture was quenched with NaHCO_3_ (10 mL) and diluted with DCM (8 mL). The organic layer were separated and aqueous layer was extracted with DCM (2 X 8 mL). The organic layers were combined and washed with brine and dried over anhydrous Na_2_SO_4_. It was filtered and concentrated in vacuum. The crude product was purified by flash column chromatography (silica gel, 20% ethyl acetate:hexanes, *v*/*v*) to give transparent/clear yellow liquid **7** (100 mg, 79%): IR (neat) 3067 (w), 2956 (s), 2930 (s), 2869 (m), 1691 (s), 1588 (m), 1571 (w), 1466 (w), 1443 (w), 1300 (s), 1279 (s), 1231 (m), 1150 (s), 1125 (m), 1045 (w), 936 (w), 751 (m), 722 (m) cm^−1^; ^1^H-NMR (400 MHz, CDCl_3_) δ 0.96 (t, *J* = 7.2 Hz, 3H), 1.35–1.43 (m, 2H), 1.46–1.59 (m, 2H), 1.61–1.72 (m, 2H), 2.22–2.33 (m, 1H), 3.28 (dd, *J* = 10, 17.6 Hz, 1H), 3.39 (dd, *J* = 3.6, 17.6 Hz, 1H), 3.58–3.67 (m, 1H), 7.76 (td, *J* = 1.2, 7.6 Hz, 1H), 7.85 (td, *J* = 1.2, 7.6 Hz, 1H), 8.07 (dd, *J* = 0.8, 7.6 Hz, 1H), 8.13 (dd, *J* = 0.8, 7.6 Hz, 1H); ^13^C-NMR (100 MHz, CDCl_3_) δ 13.7, 22.3, 25.8, 28.2, 42.0, 59.1, 124.2, 128.5, 130.5, 133.2, 135.0, 141.1, 190.7; HRMS (EI-ion trap) *m*/*z*: [M]^+^ calcd. for C_13_H_16_O_3_S, 252.0820; found 252.0823.

### 3.8. Synthesis of 3-Chloro-2-n-butyl-4H-thiochromen-4-one *(**8**)*

To a DCM solution of 2-*n*-butylthiochroman-4-one (1.0 equivalent, 0.5 mmol) was added NCS (*N*-chlorosuccinimide) (3.0 equivalent, 1.5 mmol, 200 mg) and pyridine (3.0 equivalent, 1.5 mmol, 119). The reaction mixture was stirred at room temperature for 3 h and then concentrated under vacuum to give the crude product, which was purified by flash column chromatography (silica gel, 5% ethyl acetate:hexanes, *v*/*v*) to give **8** as a white solid (78 mg, 62%): m.p. 40.5–41.3 °C; IR (neat) 3062 (w), 2959 (m), 2928 (m), 2858 (m), 1622 (s), 1567 (s), 1585 (m), 1532 (s), 1464 (m), 1437 (m), 1321 (m), 1254 (w), 1156 (w), 1098 (m), 1070 (w), 834 (m), 739 (m) cm^−1^; ^1^H-NMR (400 MHz, CDCl_3_) δ 0.92 (t, *J* = 7.2 Hz, 3H), 1.41 (sextet, *J* = 7.2 Hz, 2H), 1.65–1.74 (m, 2H), 2.80–2.86 (m, 2H), 7.45–7.59 (m, 3H), 8.48 (ddd, *J* = 0.8, 1.6, 8.0 Hz, 1H); ^13^C-NMR (100 MHz, CDCl_3_) δ 13.7, 22.5, 30.7, 36.4, 125.6, 126.5, 127.8, 129.6, 130.4, 131.5, 135.8, 150.9, 174.3; HRMS (EI-ion trap) *m*/*z*: [M]^+^ calcd. for C_13_H_13_OSCl, 252.0376; found 252.0381.

## 4. Conclusions

In conclusion, we have successfully developed the conjugate addition of lithium dialkylcuprates to thiochromones in the presence of chlorotrimethylsilane (TMSCl) and other Lewis acids, such as TMSI and TMSOTf, to afford 2-alkylthiochroman-4-ones in good yields utilizing commercially available inexpensive alkyllithium reagents. This reaction works well with simple dialkylcuprates as well as bulky dialkylcuprates (*i*-Pr_2_CuLi, *t*-Bu_2_CuLi). Lithium di-*n*-butylcuprate undergoes smooth conjugate addition to a broad range of substituted thiochromones. The 1,4-adducts (2-alkylthiochroman-4-ones) can be utilized for additional synthetic applications to access privileged sulfur-heterocycles. Further synthetic applications using these 1,4-adducts as key intermediates are ongoing in our lab.

## Figures and Tables

**Table 1:**
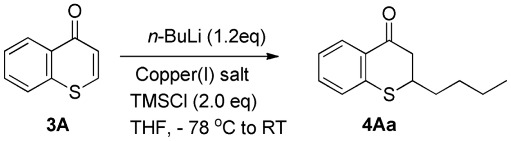
Optimization of 1,4-Conjugate Addition of Alkylcuprates to Thiochromone.

Entries ^c^	Copper (I) Salt/Reagents ^a^	Additive	% Yield ^b^
1	CuI (0.3 equiv)	none	0
2	CuCN (0.3 equiv)	none	0
3	*n*-BuCuCNLi	none	0
4	*n*-Bu_2_CuLi	none	trace
5	CuI (0.3 equiv)	TMSCl	66
6	CuCN (0.3 equiv)	TMSCl	62
7	*n*-BuCuCNLi	TMSCl	70
8	*n*-Bu_2_CuLi	TMSCl	86
9	*n*-Bu_2_CuLi	TMSI	85
10	*n*-Bu_2_CuLi	TMSOTf	82

^a^. Reagents were prepared by adding *n*-BuLi to CuCN or CuI. ^b^. Yields are based on isolated products by flash column chromatography. ^c^. Reactions were allowed to stir for 12 h and warm up to room temperature before workup.

## Supplementary Materials

The following are available online, ^1^H, and ^13^C-NMR spectra for compounds: **4Aa**, **4Ba**, **4Ca**, **4Da**, **4Ea**, **4Fa**, **4Ga**, **4Ha**, **4Ia**, **4Ja**, **5**, **6**, **7** and **8**; ^19^F-NMR spectra for compounds: **4Ha**.
