# Supplementary material for: Development of Conjugate Addition of Lithium Dialkylcuprates to Thiochromones: Synthesis of 2-Alkylthiochroman-4-ones and Additional Synthetic Applications
Source: Molecules. 2018 Jul 15;23(7):1728. doi: 10.3390/molecules23071728 (PMC6099951; doi:10.3390/molecules23071728)
Supplement: Supplementary file 1 [file molecules-23-01728-s001.pdf]

Development of conjugate addition of lithium dialkylcuprates to thiochromones: synthesis of 2-alkylthiochroman-4-ones and additional synthetic applications

Shekinah A. Bass[a], Dynasty M. Parker[a], Tania J. Bellinger[b], Aireal S. Eaton[a], Angelica S. Dibble[a], Kaata L. Koroma[b], Sylvia A. Sekyi[b], David A. Pollard[a], and Fenghai Guo[a,b,]*

[a] *Department of Chemistry, Winston Salem State University, 601 S. Martin Luther King Jr. Dr., Winston Salem, NC 27110, USA*
[b] *Biomedical Research Infrastructure Center, Winston Salem State University, Winston Salem, NC 27110, USA*

Supporting Information

Table of contents

Preparation of thiochromones S2

References S3

^{1}H, ^{13}C-NMR, and ^{19}F spectra:

^{1}H, and ^{13}C-NMR spectra for compounds: **4Aa, 4Ba**, **4Ca**, **4Da**, **4Ea**, **4Fa**, **4Ga**, **4Ha**, **4Ia**, **4Ja**, **5**, **6**, **7**, and **8** .... S4 - S19 and S21- S32

^{19}F-NMR spectra for compounds: **4Ha** S20

Preparation of starting materials – thiochromones:

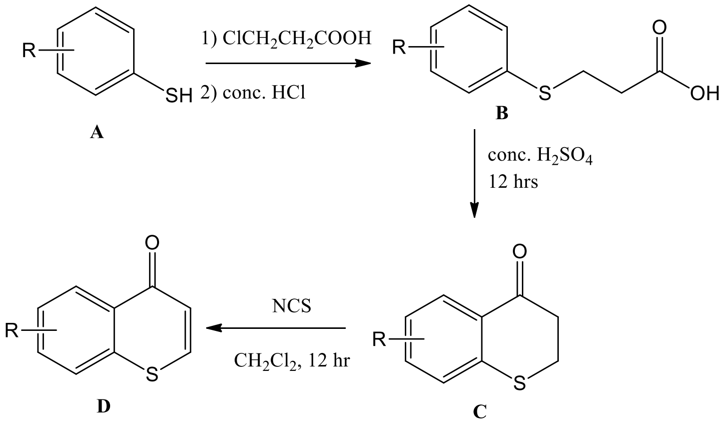

Synthesis of starting materials - thiochromones

Thiochromones **D** were prepared according to procedures reported in literature. [1,2]

Thiophenols **A** were purchased from Fisher Scientific. To a 250 mL flask with a stirrer bar, was added 1M NaOH (25 mL), 1M Na_2CO_3 (25 mL). To this, thiophenols **A** (50 mmol) was added as ethanol solution (30 mL) followed by the addition of 3-chloropropanoic acid (5.5 g, 151 mmol) in 20 mL water. The resultant reaction mixture was stirred at room temperature for 1 h, and then it was heated to reflux. When the reaction was completed as indicated by TLC monitor, the reaction mixture was cooled down to room temperature, then ethanol was evaporated under vacuum and then it was acidified to pH 1-2 with conc. HCl (18%). The solution was extracted with DCM for three times (3 X 30 mL) and the combined organic layers were dried (Na_2SO_4), filtered, and concentrated under vacuum. The crude product was then purified by flash column chromatography (EtOAc/hexanes, 5% to 25%) to give 3-(phenylthio)propanoic acid **B** in 72- 85% yield.

3-(phenylthio)propanoic acids **B** was added slowly to a 100 mL flask filled with 25 mL conc. H_2SO_4 at 0 ^oC. Then the resultant mixture was warmed up to room temperature and stirred at room temperature for about 12 hrs. When the reaction is finished as indicated by TLC monitoring, the reaction mixture was poured onto ice and extracted with CH_2Cl_2 (3X 30 mL)upon warming up to room temperature. The organic layers were combined and dried over anhydrous Na_2SO_4. It was then filtered, and concentrated under vacuum. The crude product was purified by flash column chromatography (EtOAc/hexanes, 10%) to afford thiochroman-4-one **C** in 60-80% yield.

To a cool solution (ice bath) of **C** (10 mmol) in CH_2Cl_2 (30 ml) was added *N*-chlorosuccinimide (NCS) (10.5 mmol, 1.05 equivalent) in one portion. The resultant mixture was then warmed up to room temperature and stirred overnight for about 12 hours. It was quenched with water (30 ml) and the organic layer was separated. The aqueous layer was extracted with DCM (2 X 30 mL). The organic layers were combined and dried (Na_2SO_4). It was then filtered, concentrated under vacuum to give crude product. The crude product was then purified by flash column chromatography (EtOAc/hexanes, 5-10%) to give thiochromone **D** in 50-70% yield.

References:

1. Giles, P. R.; Marson, C. M. *Aust. J. Chem*. **1992**, 45, 439–443.

2. Jia, W.; Liu, Y.-J.; Li, W.; Liu, Y.; Zhang, D.-J.; Zhang, P.; Gong, P. *Bioorg. Med. Chem*. **2009**, 17, 4569–4574.

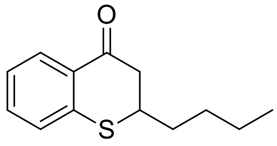

4Aa

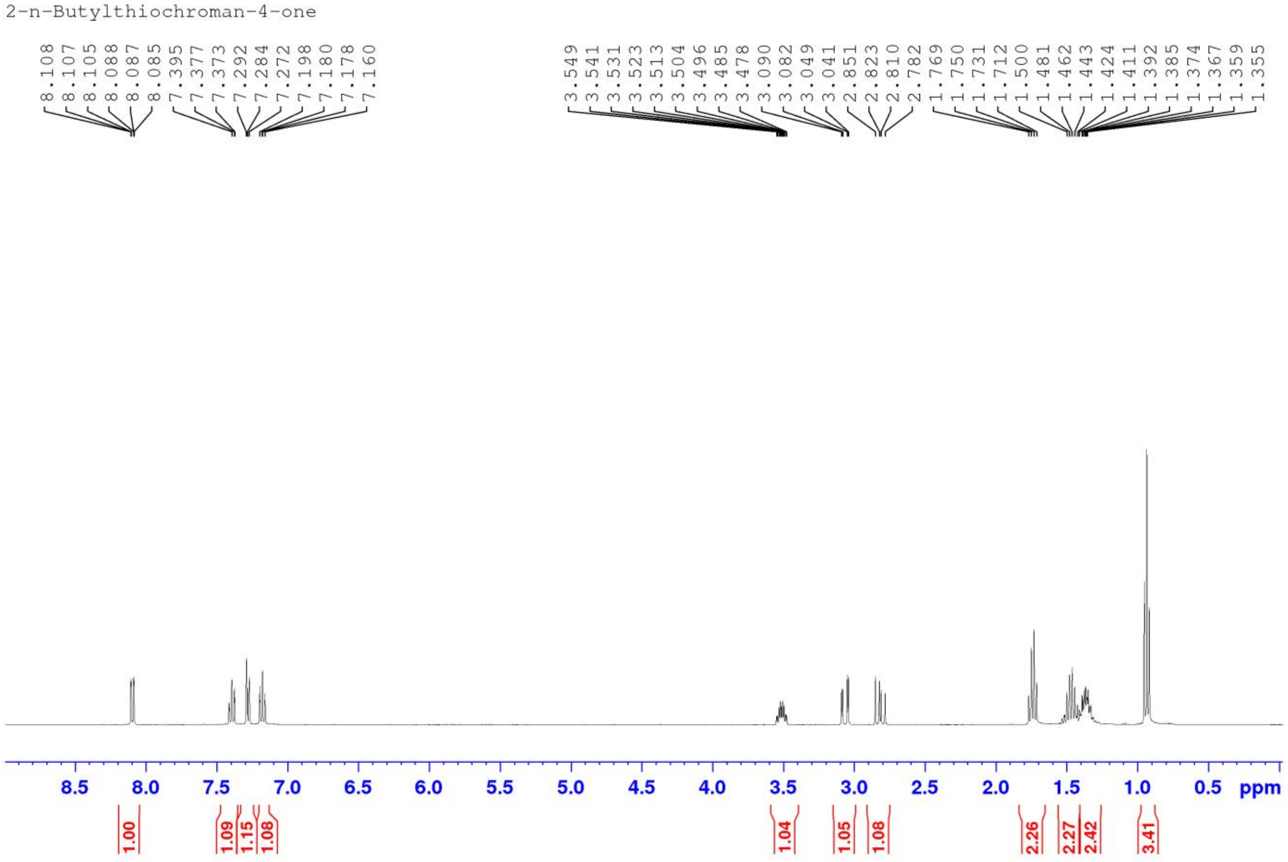

2-n-Butylthiochroman-4-one
8.108 8.107 8.105 8.088 8.087 8.085 7.395 7.377 7.373 7.292 7.284 7.272 7.198 7.180 7.178 7.160
3.549 3.541 3.531 3.523 3.513 3.504 3.496 3.485 3.478 3.090 3.082 3.049 3.041 2.851 2.823 2.810 2.782 1.769 1.750 1.731 1.712 1.500 1.481 1.462 1.443 1.424 1.411 1.392 1.385 1.374 1.367 1.359 1.355
8.5 8.0 7.5 7.0 6.5 6.0 5.5 5.0 4.5 4.0 3.5 3.0 2.5 2.0 1.5 1.0 0.5 ppm
1.00 1.09 1.15 1.08 1.04 1.05 1.08 2.26 2.27 2.42 3.41

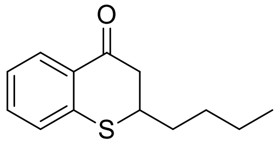

4Aa

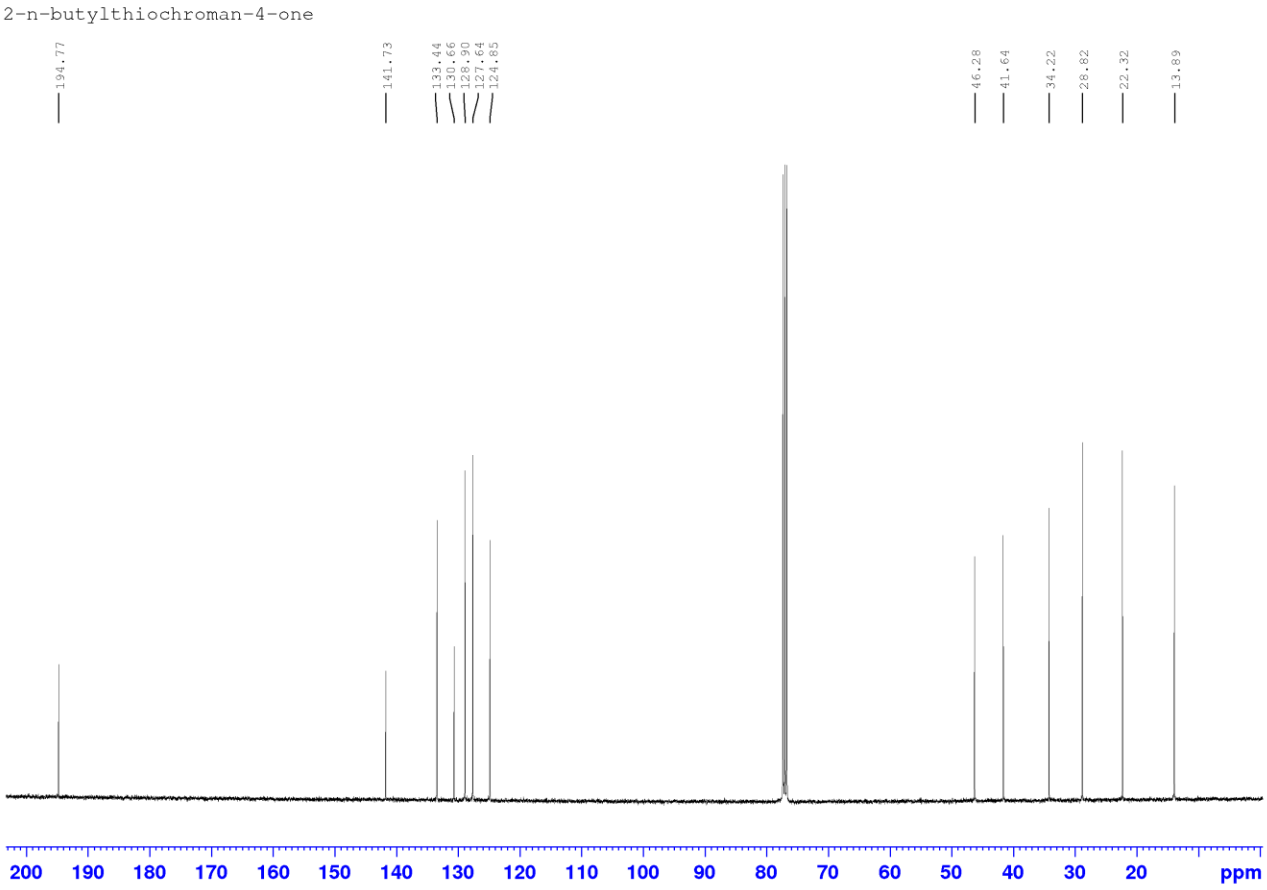
2-n-butylthiochroman-4-one
194.77
141.73
133.44
130.66
128.90
127.64
124.85
46.28
41.64
34.22
28.82
22.32
13.89
200 190 180 170 160 150 140 130 120 110 100 90 80 70 60 50 40 30 20 ppm

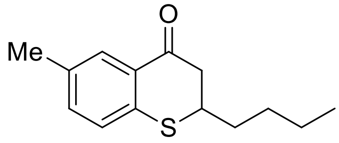

4Ba

6-methyl-2-n-butylthiochroman-4-one

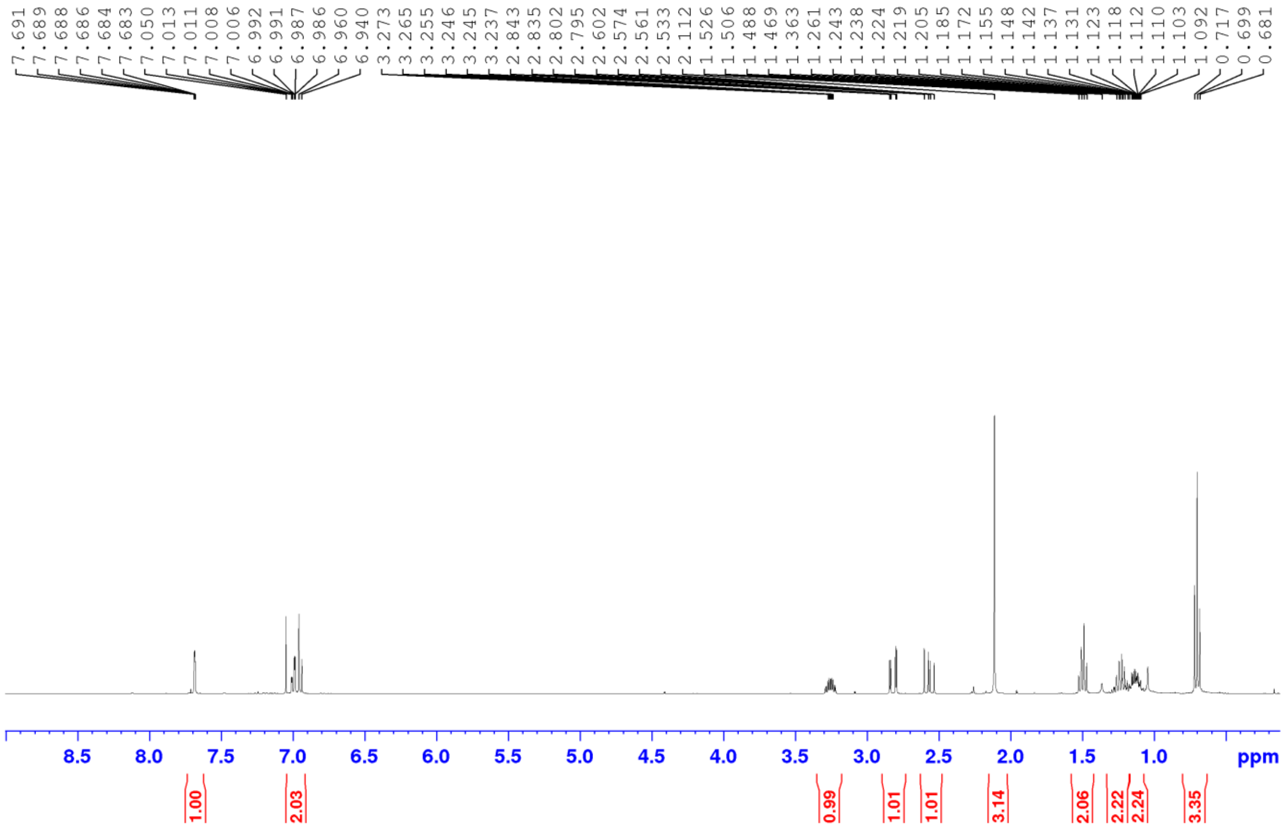

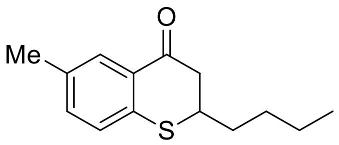
Me
O
S

4Ba

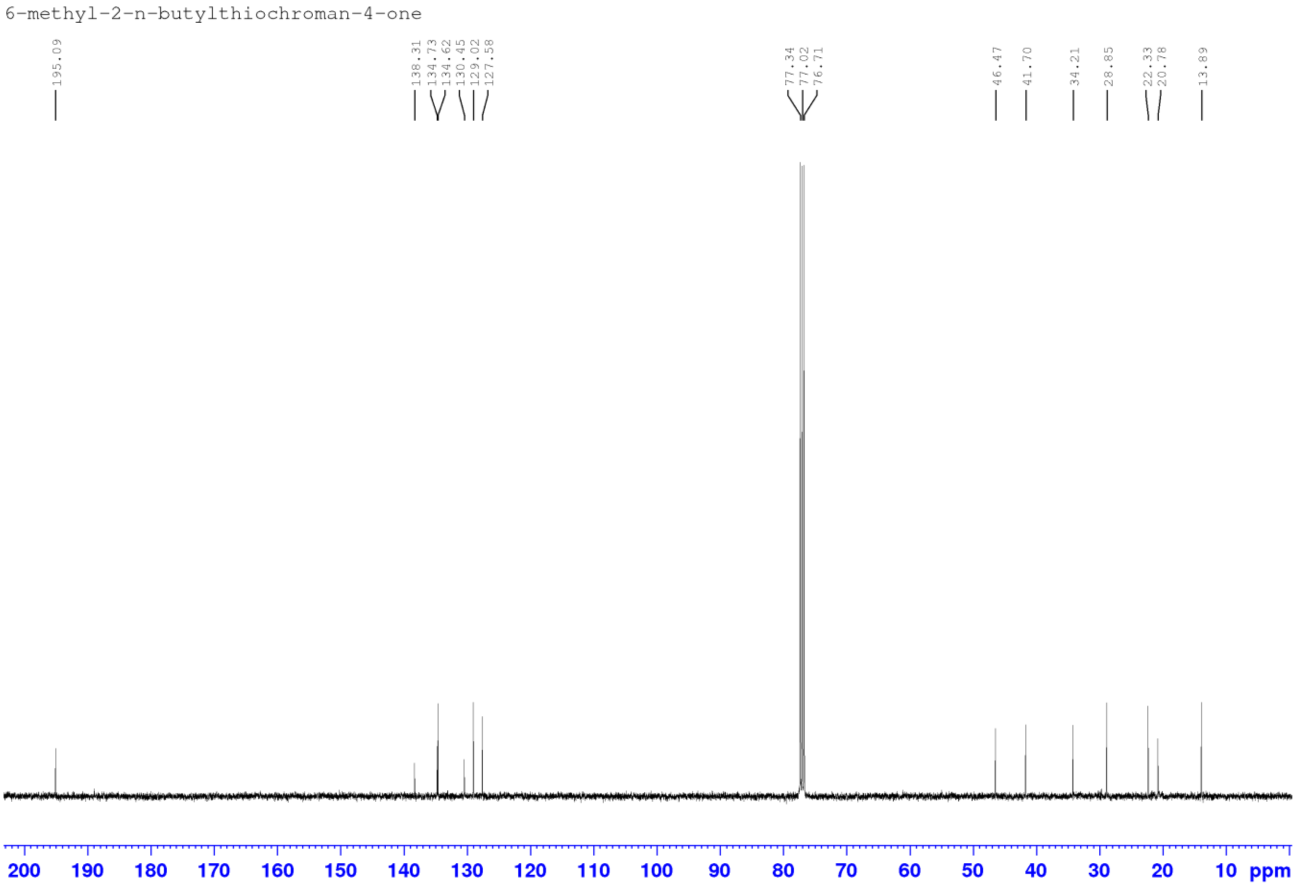
6-methyl-2-n-butylthiochroman-4-one
195.09
138.31
134.73
134.62
130.45
129.02
127.58
77.34
77.02
76.71
46.47
41.70
34.21
28.85
22.33
20.78
13.89
200
190
180
170
160
150
140
130
120
110
100
90
80
70
60
50
40
30
20
10
ppm

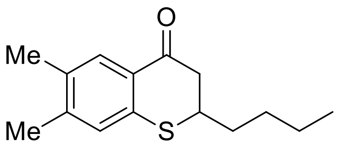

4Ca

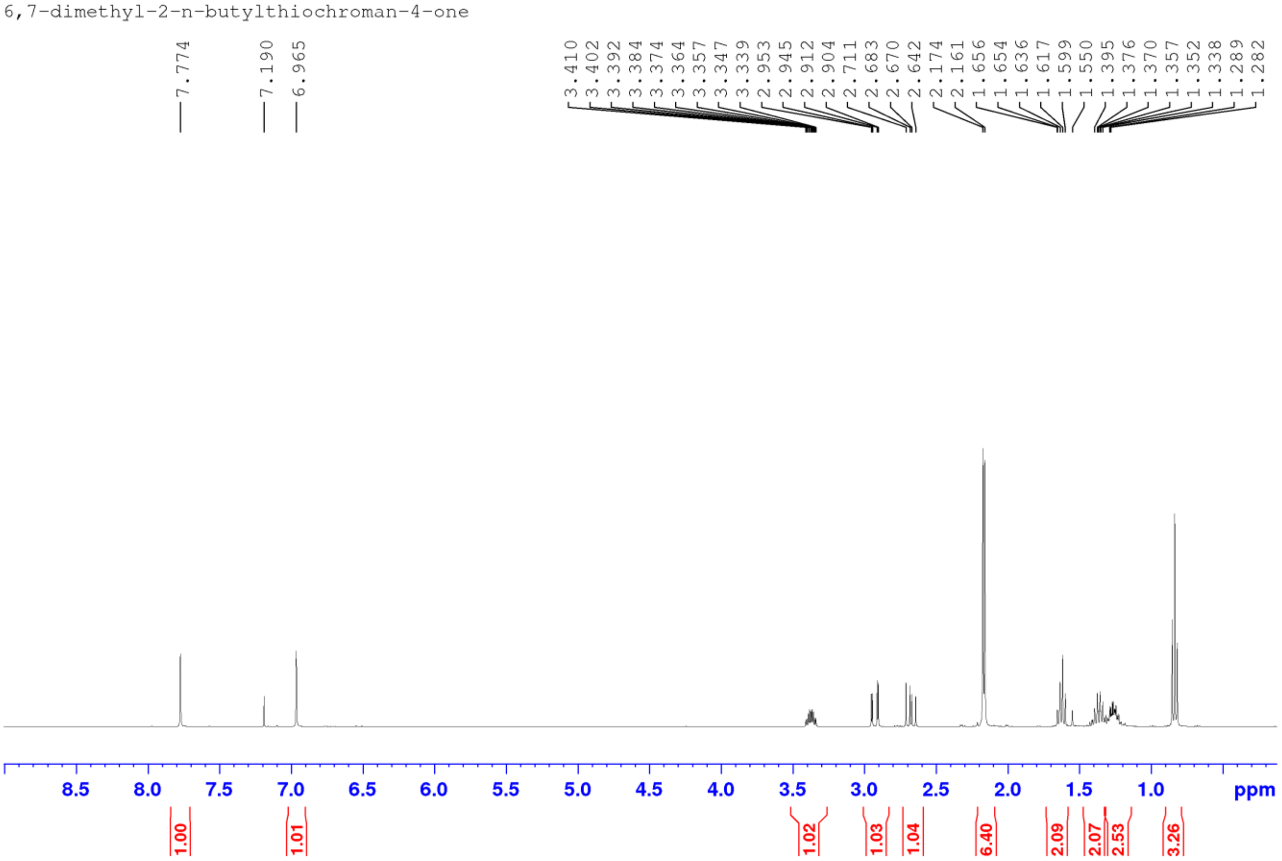
6,7-dimethyl-2-n-butylthiochroman-4-one
7.774
7.190
6.965
3.410
3.402
3.392
3.384
3.374
3.364
3.357
3.347
3.339
2.953
2.945
2.912
2.904
2.711
2.683
2.670
2.642
2.174
2.161
1.656
1.654
1.636
1.617
1.599
1.550
1.395
1.376
1.370
1.357
1.352
1.338
1.289
1.282
8.5
8.0
7.5
7.0
6.5
6.0
5.5
5.0
4.5
4.0
3.5
3.0
2.5
2.0
1.5
1.0
ppm
1.00
1.01
1.02
1.03
1.04
6.40
2.09
2.07
2.53
3.26

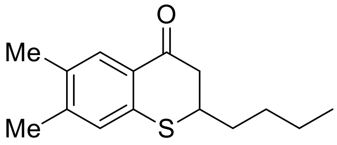
O
Me
Me
S

4Ca

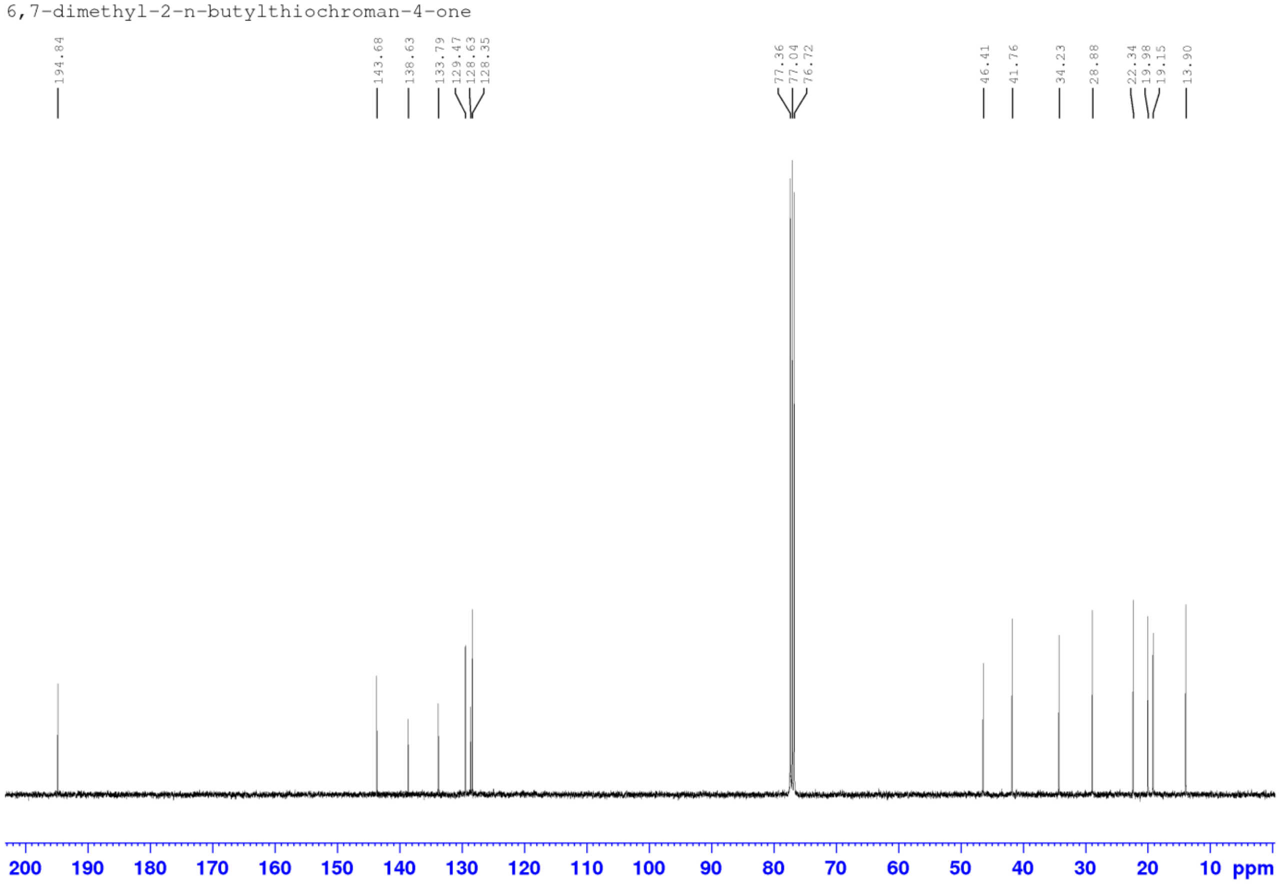
6,7-dimethyl-2-n-butylthiochroman-4-one
194.84
143.68
138.63
133.79
129.47
128.63
128.35
77.36
77.04
76.72
46.41
41.76
34.23
28.88
22.34
19.98
19.15
13.90
200
190
180
170
160
150
140
130
120
110
100
90
80
70
60
50
40
30
20
10
ppm

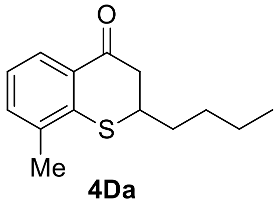
O
S
Me

4Da

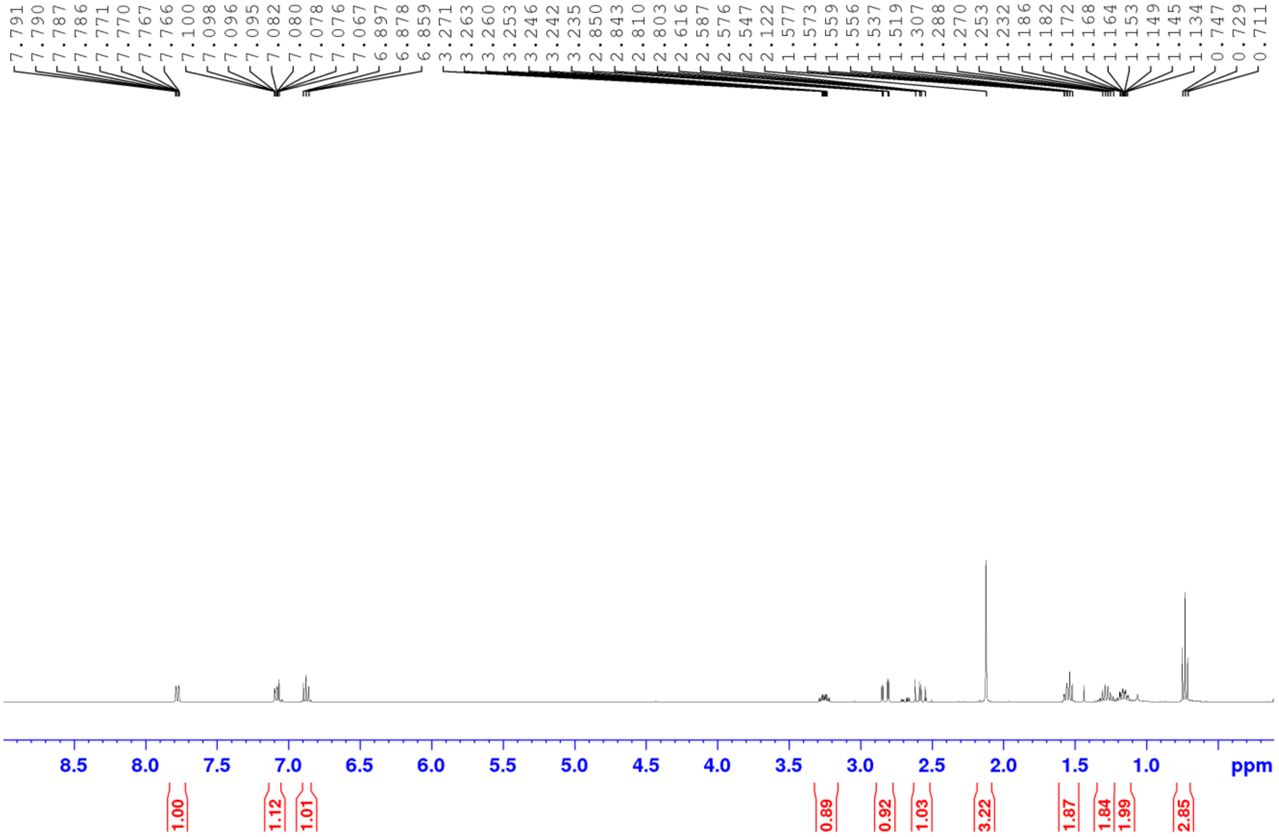
7.791
7.790
7.787
7.786
7.771
7.770
7.767
7.766
7.100
7.098
7.096
7.095
7.082
7.080
7.078
7.076
7.067
6.897
6.878
6.859
3.271
3.263
3.260
3.253
3.246
3.242
3.235
2.850
2.843
2.810
2.803
2.616
2.587
2.576
2.547
2.122
1.577
1.573
1.559
1.556
1.537
1.519
1.307
1.288
1.270
1.253
1.232
1.186
1.182
1.172
1.168
1.164
1.153
1.149
1.145
1.134
0.747
0.729
0.711
8.5
8.0
7.5
7.0
6.5
6.0
5.5
5.0
4.5
4.0
3.5
3.0
2.5
2.0
1.5
1.0
ppm
1.00
1.12
1.01
0.89
0.92
1.03
3.22
1.87
1.84
1.99
2.85

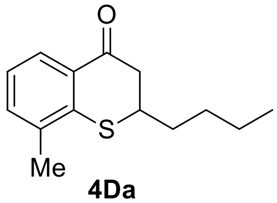

O
S
Me

4Da

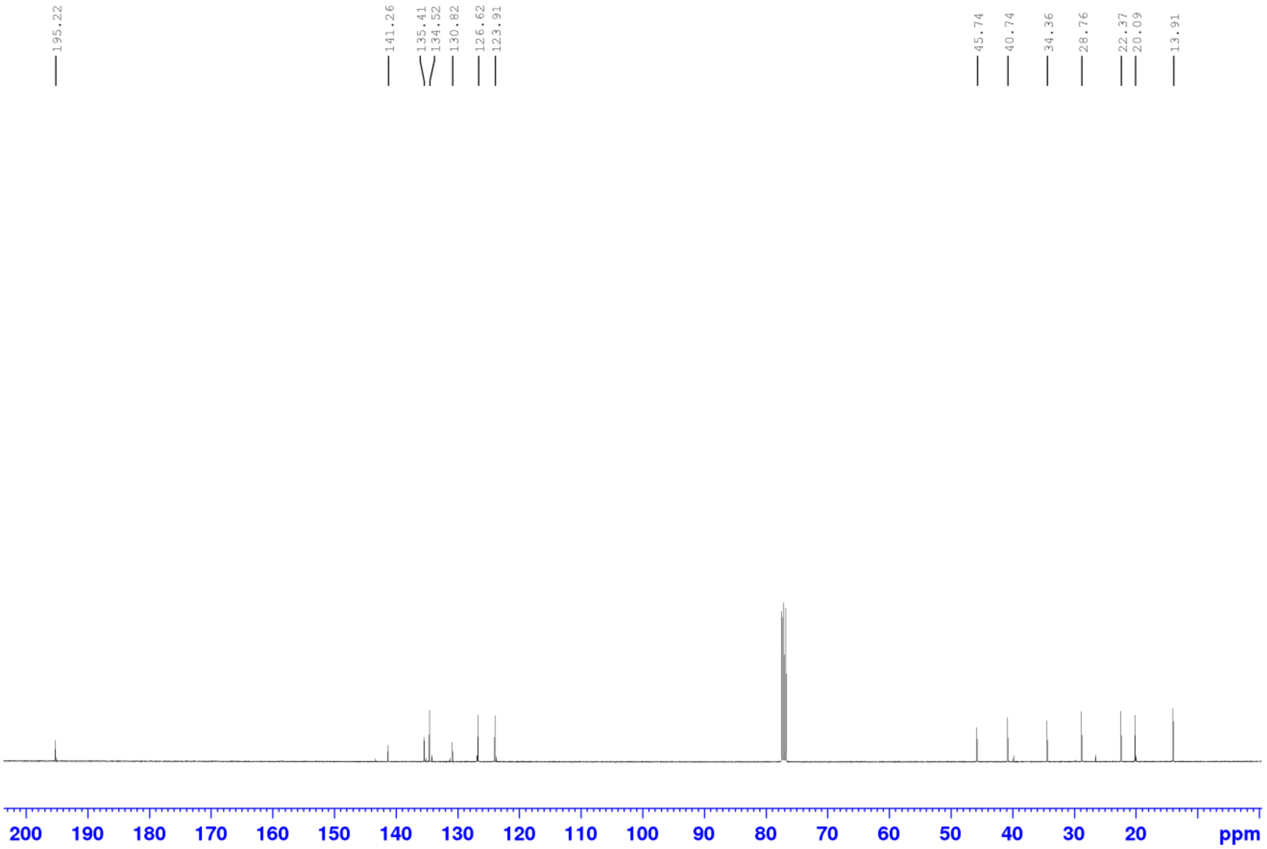

195.22
141.26
135.41
134.52
130.82
126.62
123.91
45.74
40.74
34.36
28.76
22.37
20.09
13.91
200
190
180
170
160
150
140
130
120
110
100
90
80
70
60
50
40
30
20
ppm

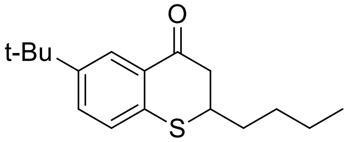
O
t-Bu
S

4Ea

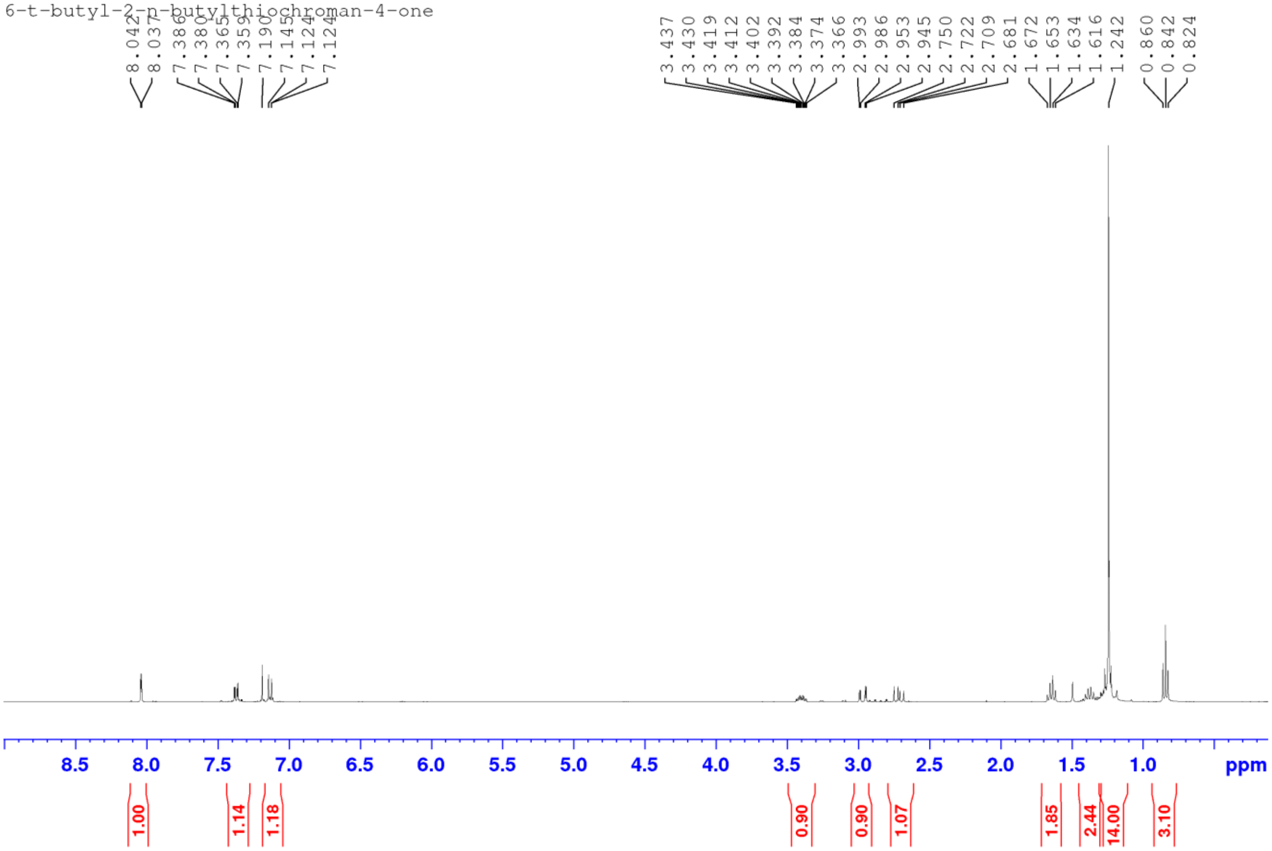
6-t-butyl-2-n-butylthiochroman-4-one
8.042
8.037
7.386
7.380
7.365
7.359
7.190
7.145
7.124
7.124
3.437
3.430
3.419
3.412
3.402
3.392
3.384
3.374
3.366
2.993
2.986
2.953
2.945
2.750
2.722
2.709
2.681
1.672
1.653
1.634
1.616
1.242
0.860
0.842
0.824
8.5
8.0
7.5
7.0
6.5
6.0
5.5
5.0
4.5
4.0
3.5
3.0
2.5
2.0
1.5
1.0
ppm
1.00
1.14
1.18
0.90
0.90
1.07
1.85
2.44
14.00
3.10

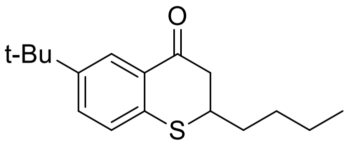

4Ea

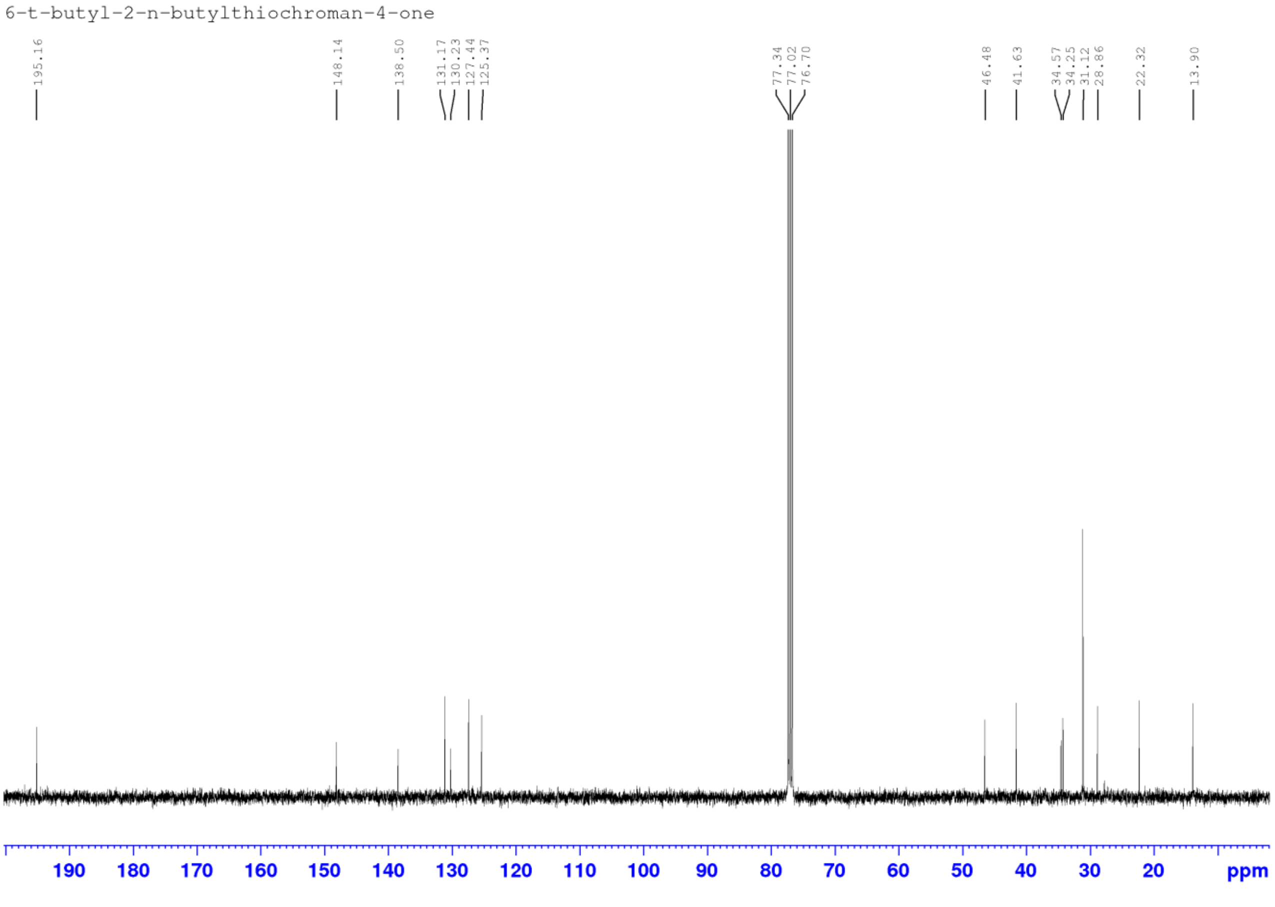
6-t-butyl-2-n-butylthiochroman-4-one
195.16
148.14
138.50
131.17
130.23
127.44
125.37
77.34
77.02
76.70
46.48
41.63
34.57
34.25
31.12
28.86
22.32
13.90
190 180 170 160 150 140 130 120 110 100 90 80 70 60 50 40 30 20 ppm

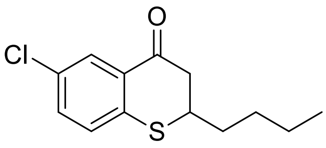

4Fa

6-chloro-2-n-butylthiochroman-4-one

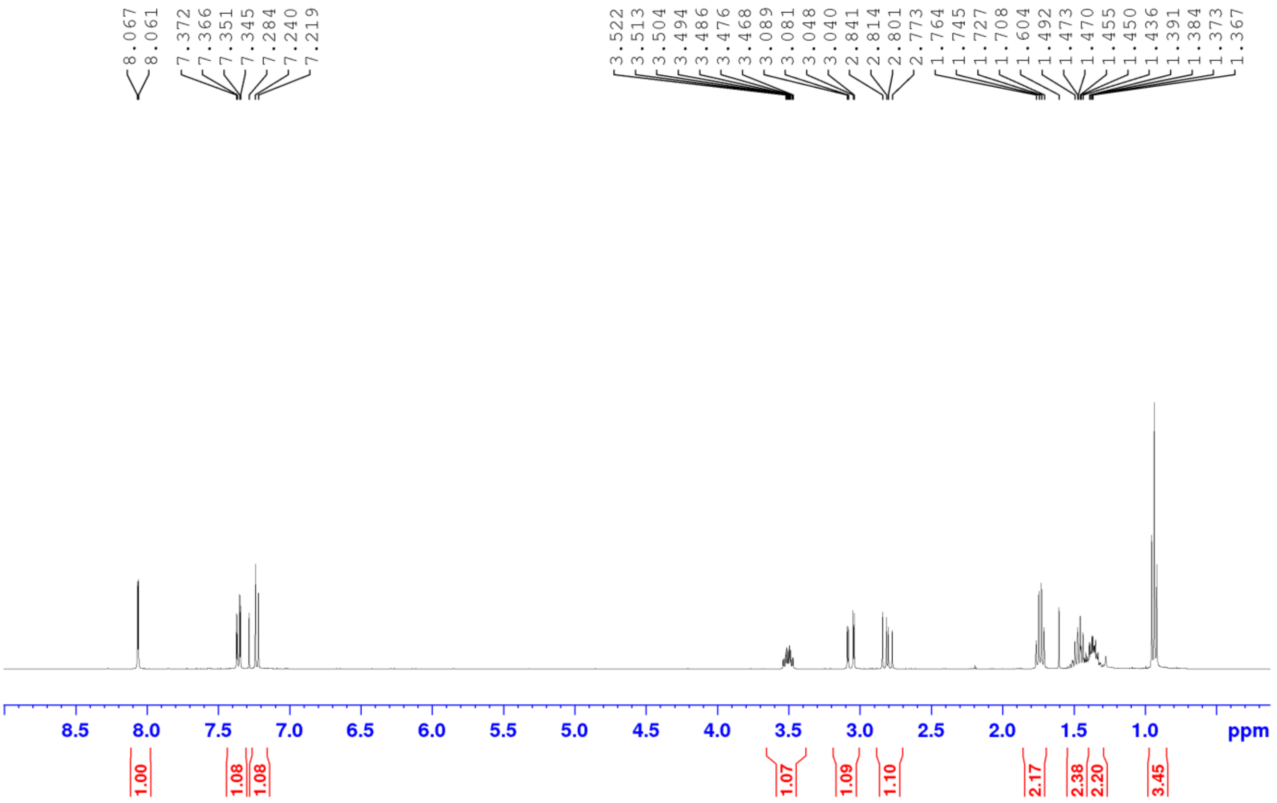

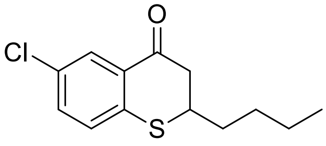
O
Cl
S

4Fa

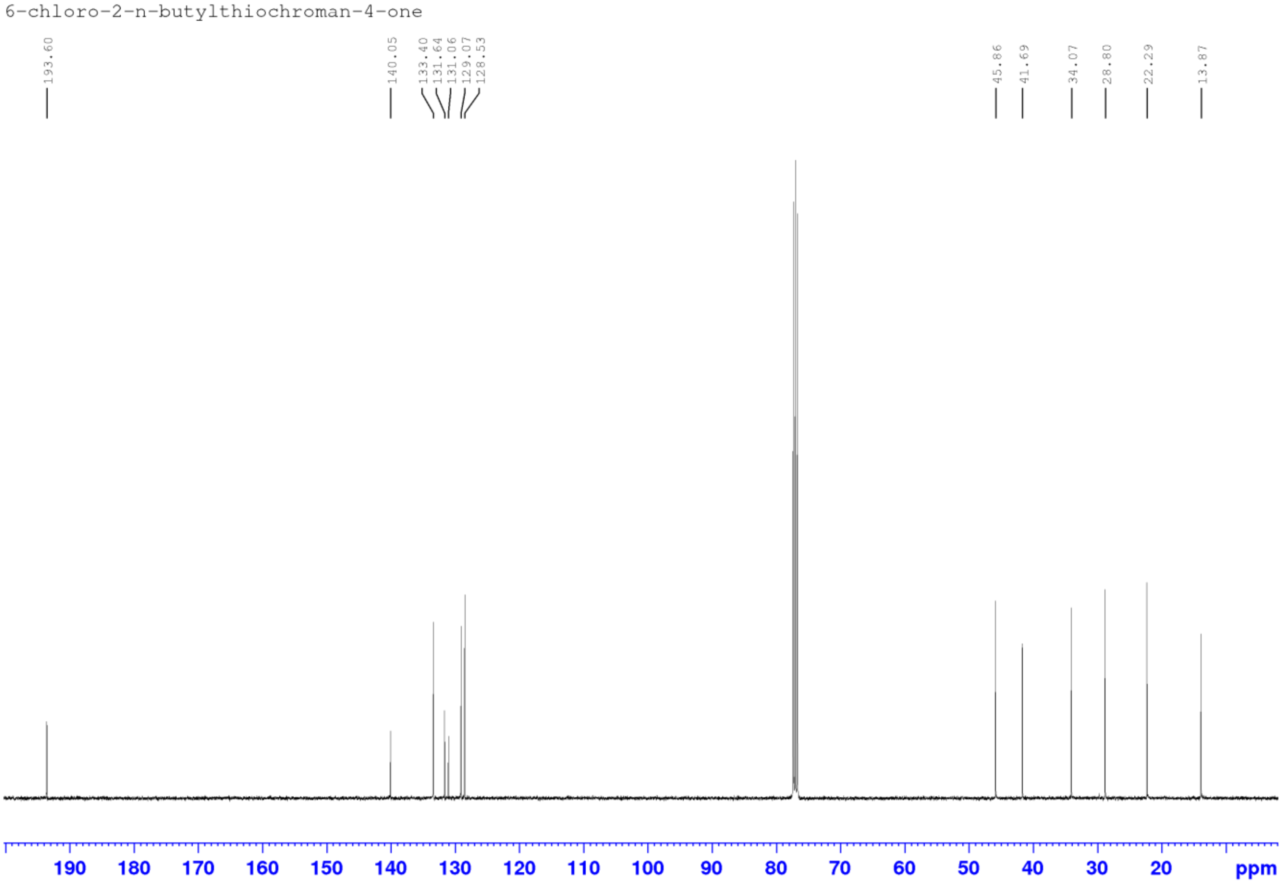
6-chloro-2-n-butylthiochroman-4-one
193.60
140.05
133.40
131.64
131.06
129.07
128.53
45.86
41.69
34.07
28.80
22.29
13.87
190
180
170
160
150
140
130
120
110
100
90
80
70
60
50
40
30
20
ppm

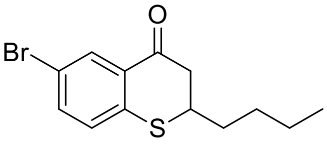

4Ga

6-bromo-2-n-butylthiochroman-4-one

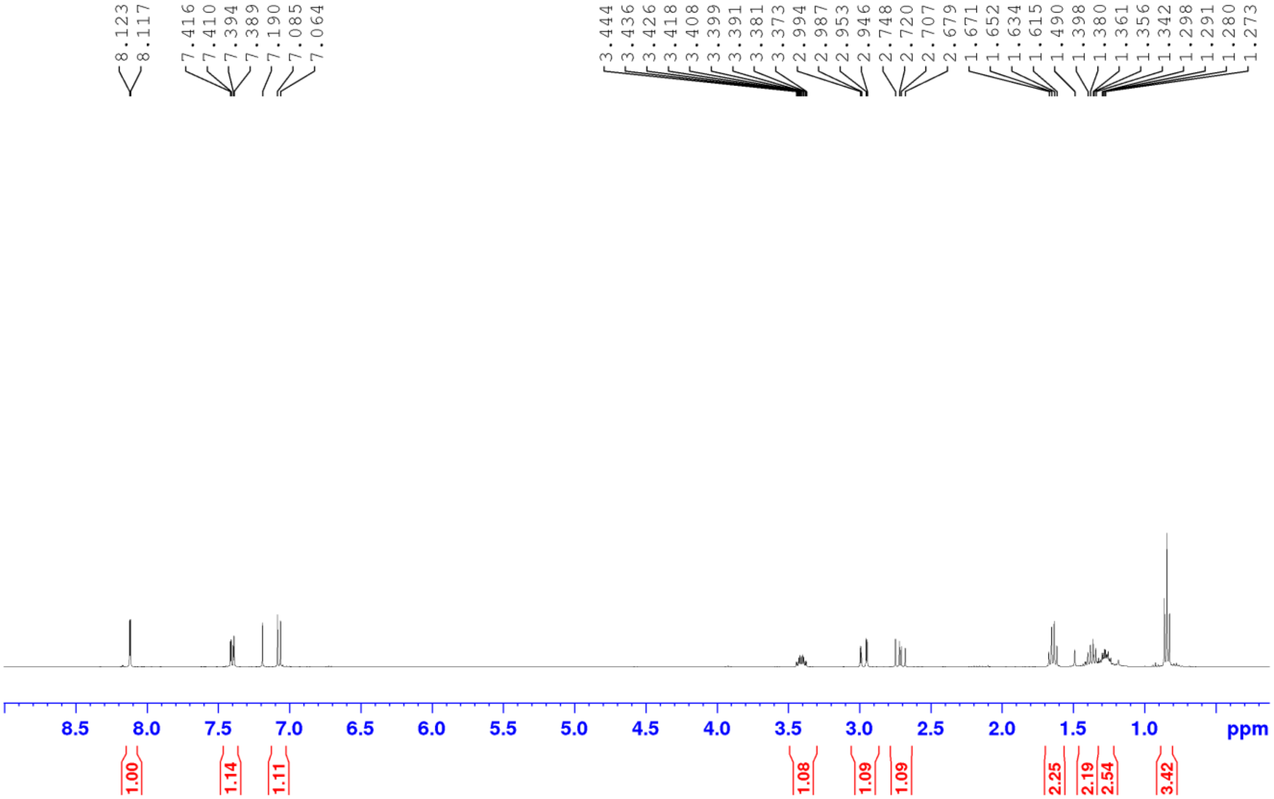

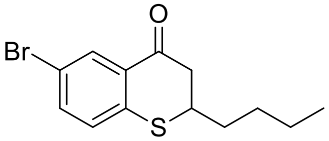
O
Br
S

4Ga

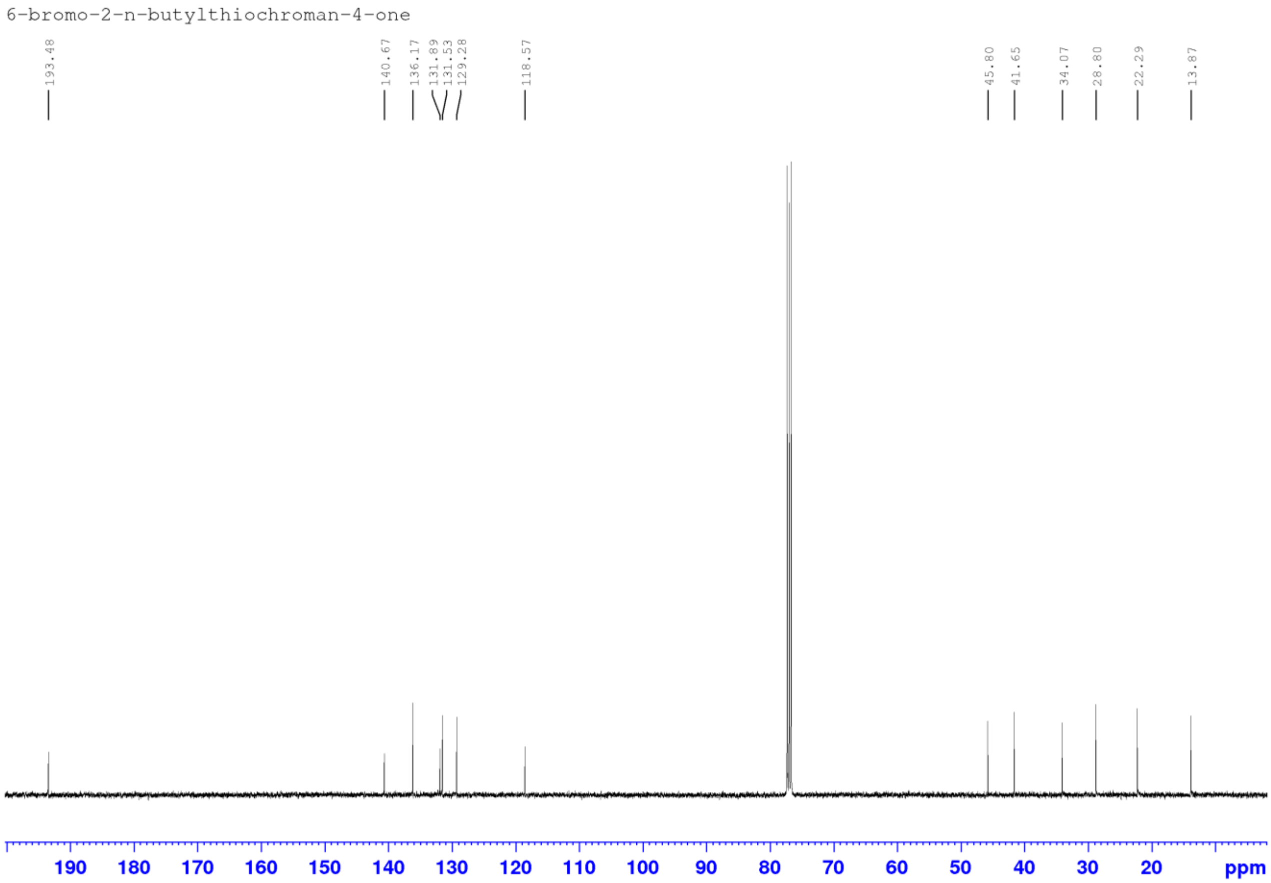
6-bromo-2-n-butylthiochroman-4-one
193.48
140.67
136.17
131.89
131.53
129.28
118.57
45.80
41.65
34.07
28.80
22.29
13.87
190
180
170
160
150
140
130
120
110
100
90
80
70
60
50
40
30
20
ppm

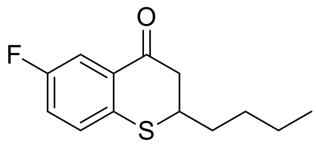

4Ha

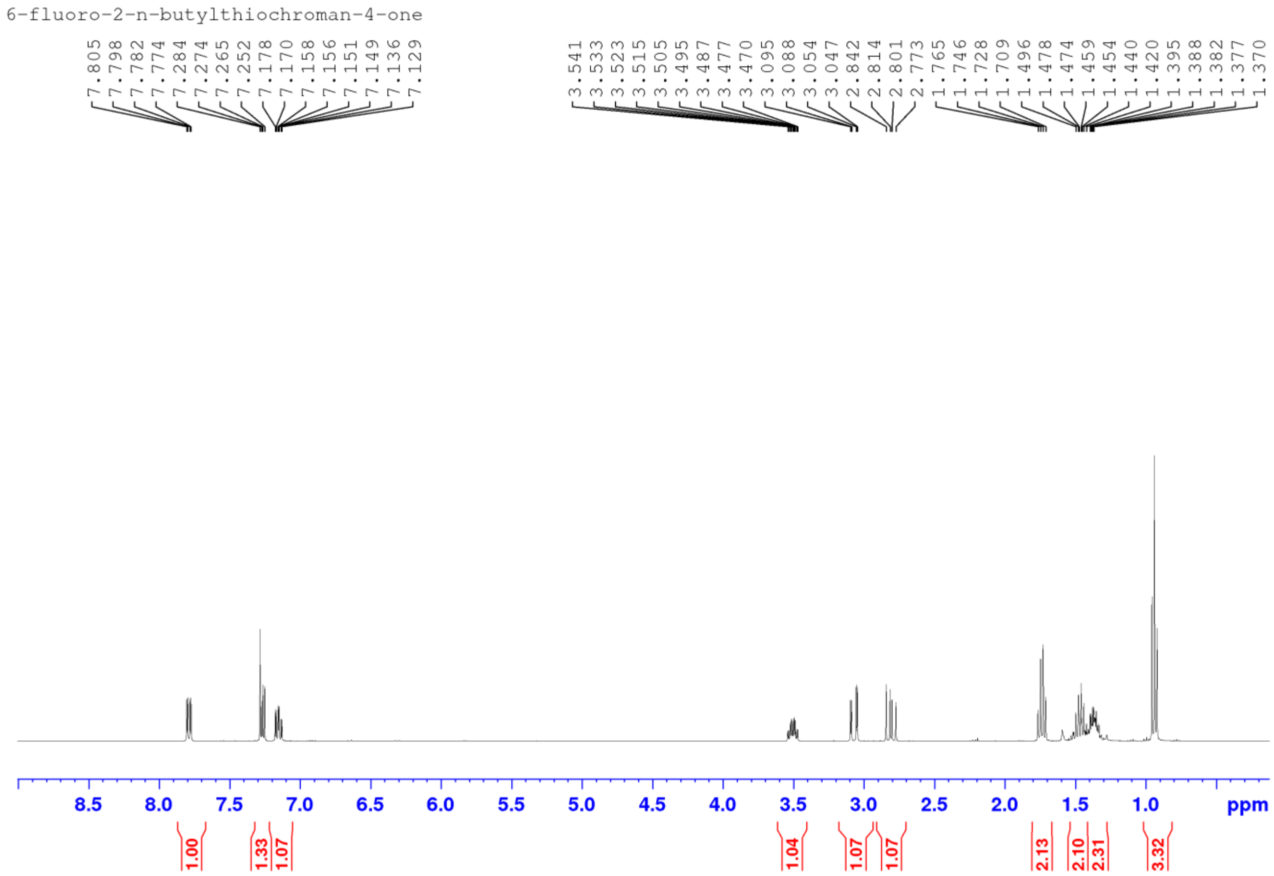
6-fluoro-2-n-butylthiochroman-4-one
7.805
7.798
7.782
7.774
7.284
7.274
7.265
7.252
7.178
7.170
7.158
7.156
7.151
7.149
7.136
7.129
3.541
3.533
3.523
3.515
3.505
3.495
3.487
3.477
3.470
3.095
3.088
3.054
3.047
2.842
2.814
2.801
2.773
1.765
1.746
1.728
1.709
1.496
1.478
1.474
1.459
1.454
1.440
1.420
1.395
1.388
1.382
1.377
1.370
8.5
8.0
7.5
7.0
6.5
6.0
5.5
5.0
4.5
4.0
3.5
3.0
2.5
2.0
1.5
1.0
ppm
1.00
1.33
1.07
1.04
1.07
1.07
2.13
2.10
2.31
3.32

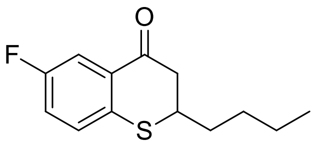
O
F
S

4Ha

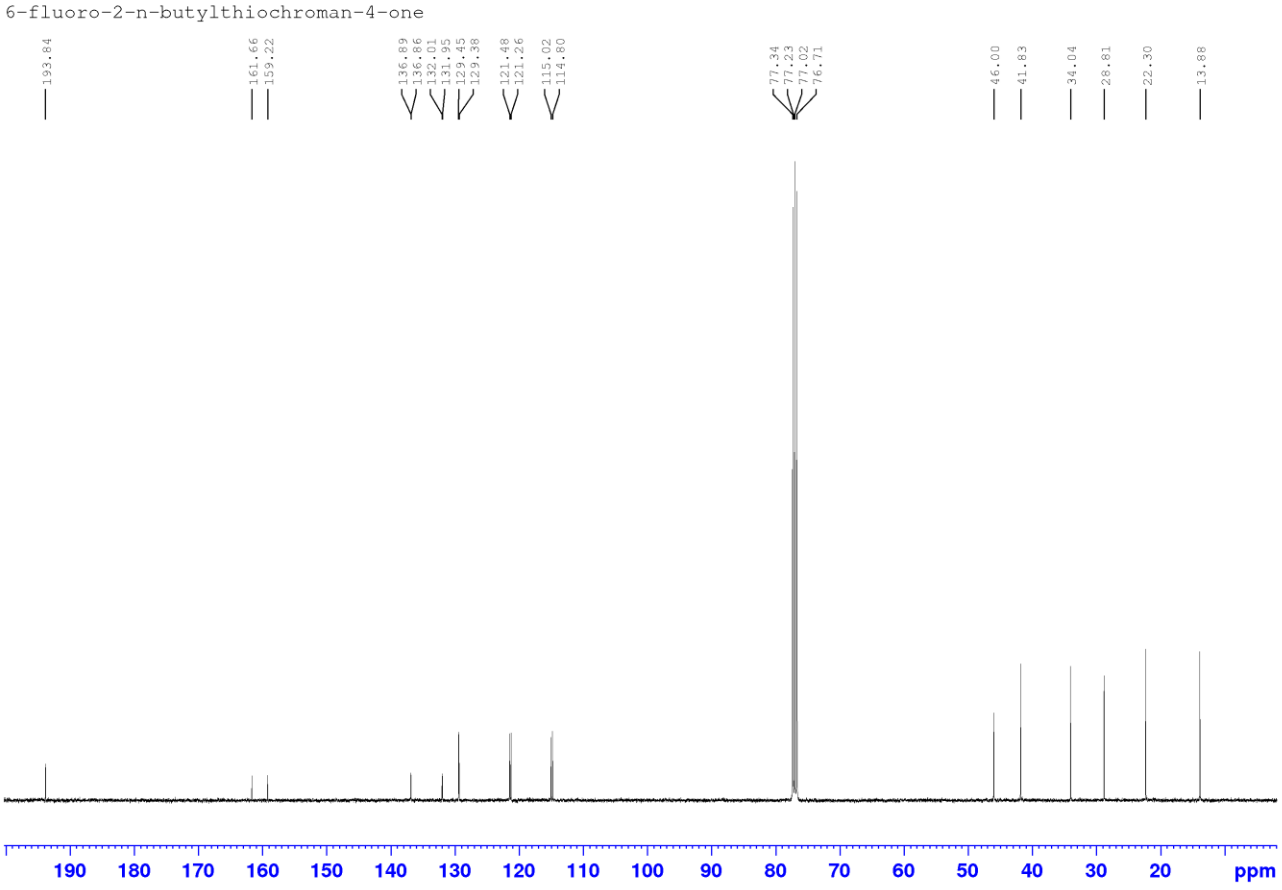
6-fluoro-2-n-butylthiochroman-4-one
193.84
161.66
159.22
136.89
136.86
132.01
131.95
129.45
129.38
121.48
121.26
115.02
114.80
77.34
77.23
77.02
76.71
46.00
41.83
34.04
28.81
22.30
13.88
190
180
170
160
150
140
130
120
110
100
90
80
70
60
50
40
30
20
ppm

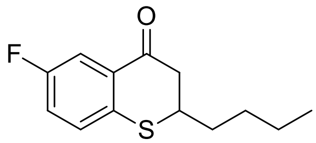

4Ha

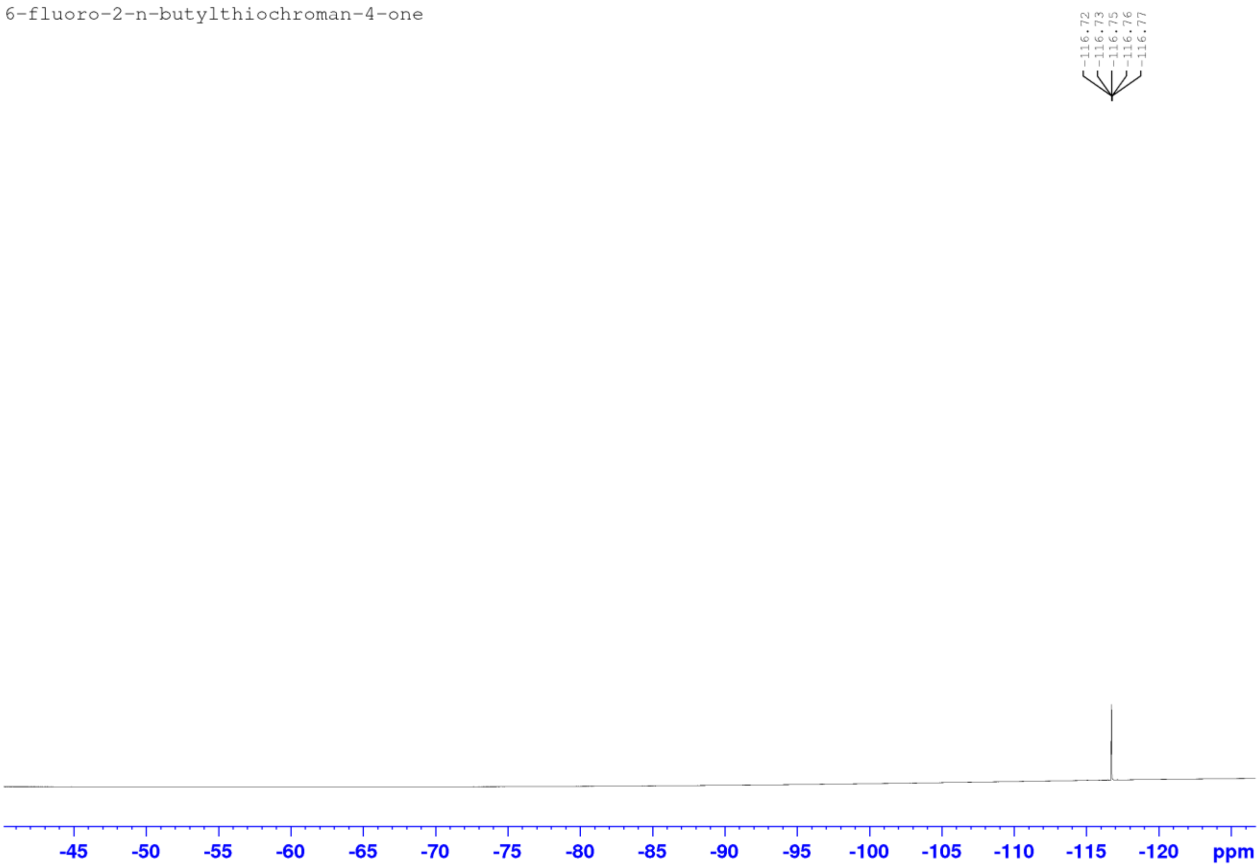

6-fluoro-2-n-butylthiochroman-4-one
-116.72
-116.73
-116.75
-116.76
-116.77
-45
-50
-55
-60
-65
-70
-75
-80
-85
-90
-95
-100
-105
-110
-115
-120
ppm

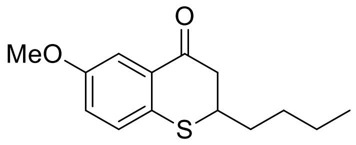

4la

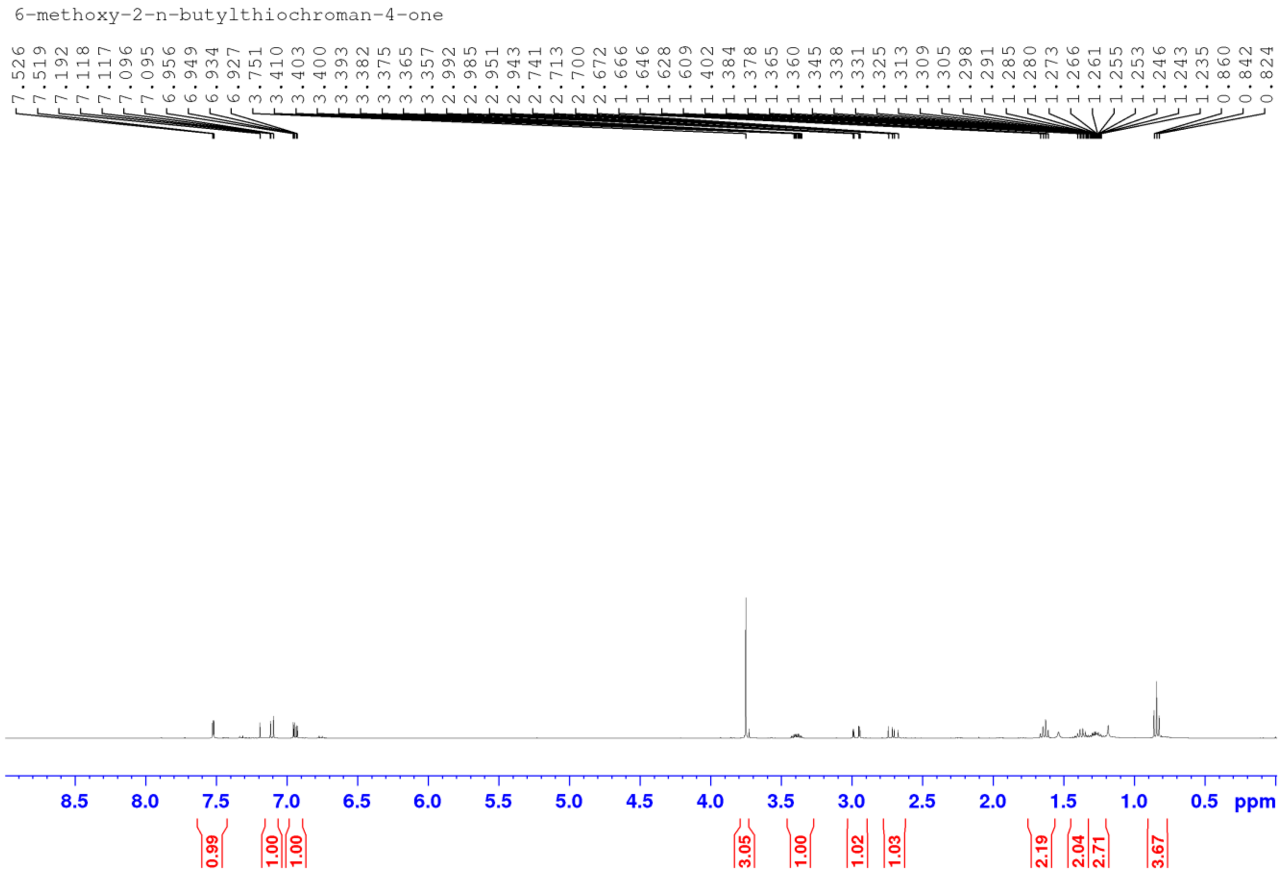
6-methoxy-2-n-butylthiochroman-4-one
7.526
7.519
7.192
7.118
7.117
7.096
7.095
6.956
6.949
6.934
6.927
3.751
3.410
3.403
3.400
3.393
3.382
3.375
3.365
3.357
2.992
2.985
2.951
2.943
2.741
2.713
2.700
2.672
1.666
1.646
1.628
1.609
1.402
1.384
1.378
1.365
1.360
1.345
1.338
1.331
1.325
1.313
1.309
1.305
1.298
1.291
1.285
1.280
1.273
1.266
1.261
1.255
1.253
1.246
1.243
1.235
0.860
0.842
0.824
8.5
8.0
7.5
7.0
6.5
6.0
5.5
5.0
4.5
4.0
3.5
3.0
2.5
2.0
1.5
1.0
0.5
ppm
0.99
1.00
1.00
3.05
1.00
1.02
1.03
2.19
2.04
2.71
3.67

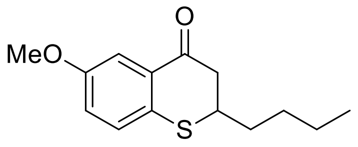

MeO
O
S

4la

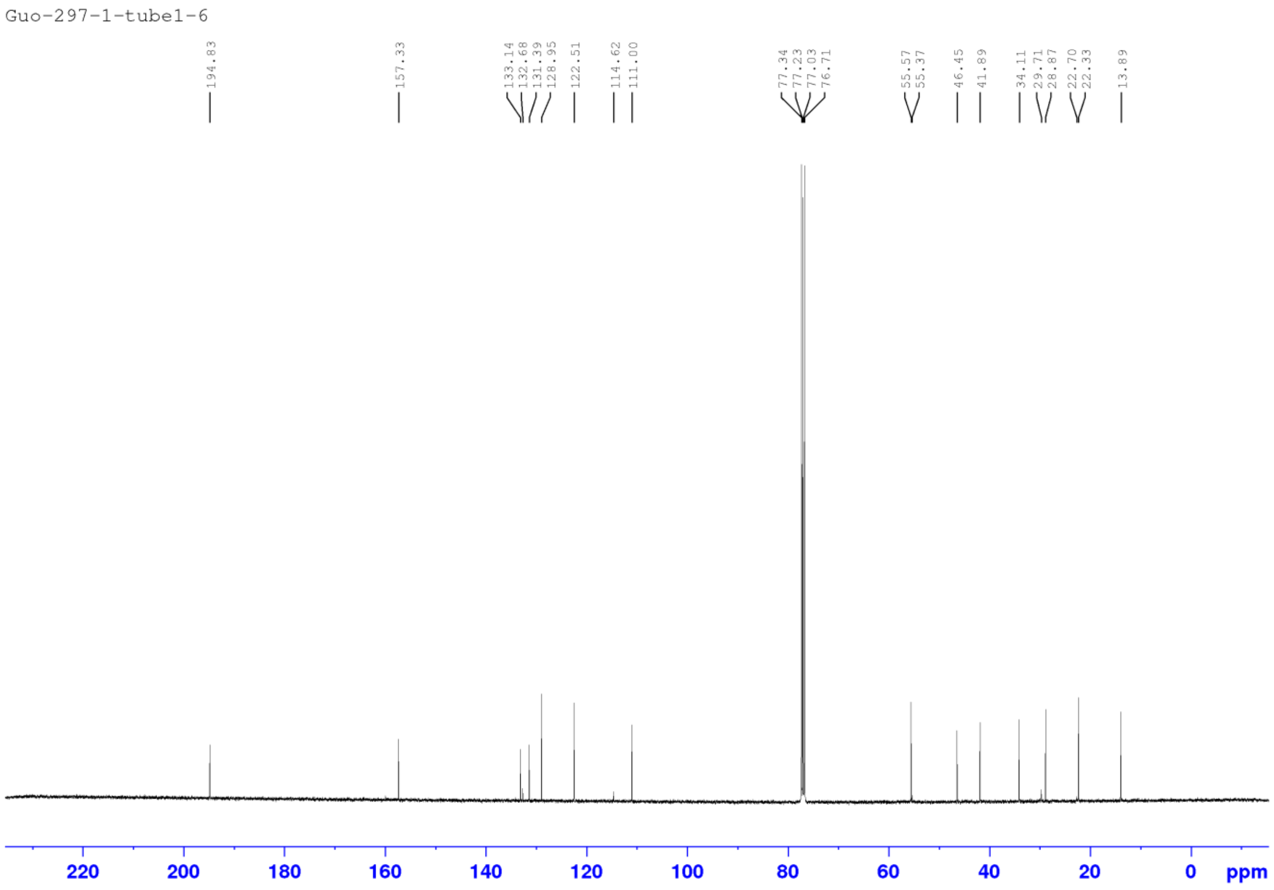

Guo-297-1-tube1-6
194.83
157.33
133.14
132.68
131.39
128.95
122.51
114.62
111.00
77.34
77.23
77.03
76.71
55.57
55.37
46.45
41.89
34.11
29.71
28.87
22.70
22.33
13.89
220
200
180
160
140
120
100
80
60
40
20
0
ppm

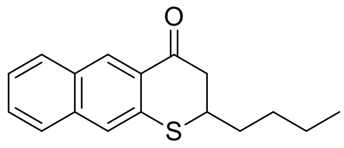

4Ja

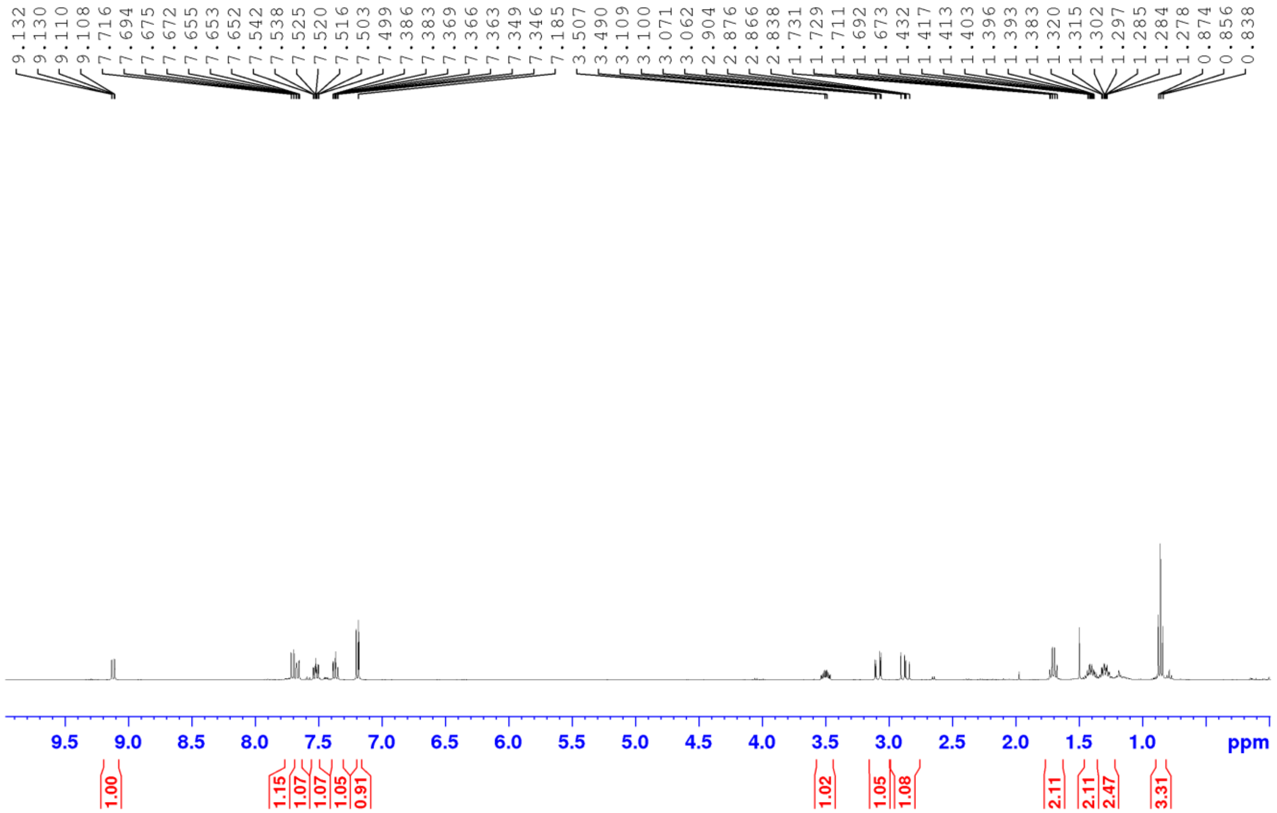
9.132 9.130 9.110 9.108 7.716 7.694 7.675 7.672 7.655 7.653 7.652 7.542 7.538 7.525 7.520 7.516 7.503 7.499 7.386 7.383 7.369 7.366 7.363 7.349 7.346 7.185 3.507 3.490 3.109 3.100 3.071 3.062 2.904 2.876 2.866 2.838 1.731 1.729 1.711 1.692 1.673 1.432 1.417 1.413 1.403 1.396 1.393 1.383 1.320 1.315 1.302 1.297 1.285 1.284 1.278 0.874 0.856 0.838
9.5 9.0 8.5 8.0 7.5 7.0 6.5 6.0 5.5 5.0 4.5 4.0 3.5 3.0 2.5 2.0 1.5 1.0 ppm
1.00 1.15 1.07 1.07 1.05 0.91 1.02 1.05 1.08 2.11 2.11 2.47 3.31

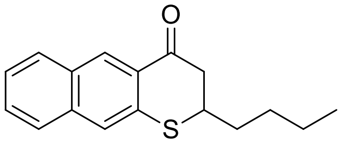
O
S

4Ja

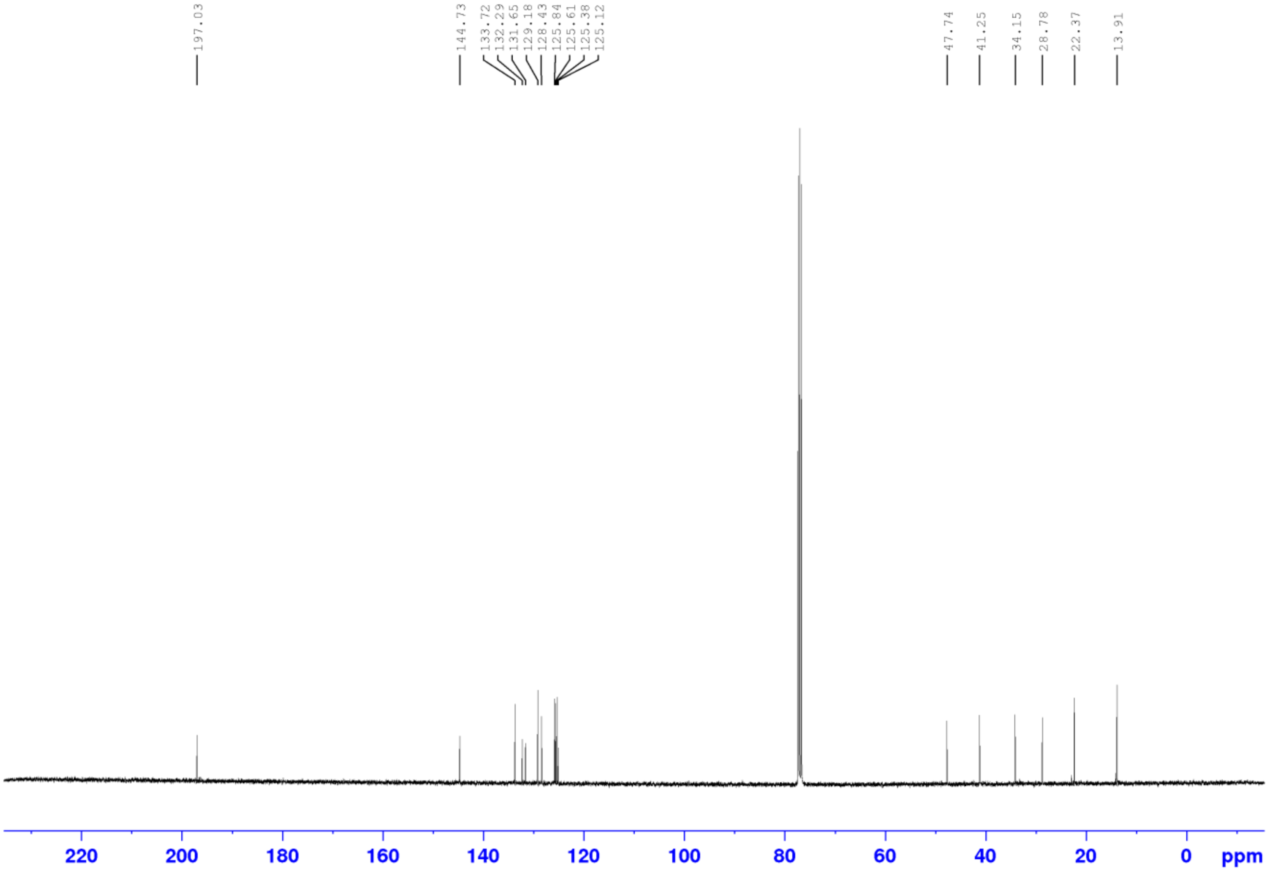
197.03
144.73
133.72
132.29
131.65
129.18
128.43
125.84
125.61
125.38
125.12
47.74
41.25
34.15
28.78
22.37
13.91
220
200
180
160
140
120
100
80
60
40
20
0
ppm

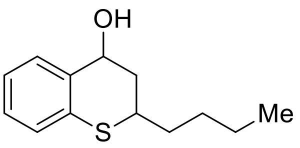

5

2-n-butylthiochrman-4-ol

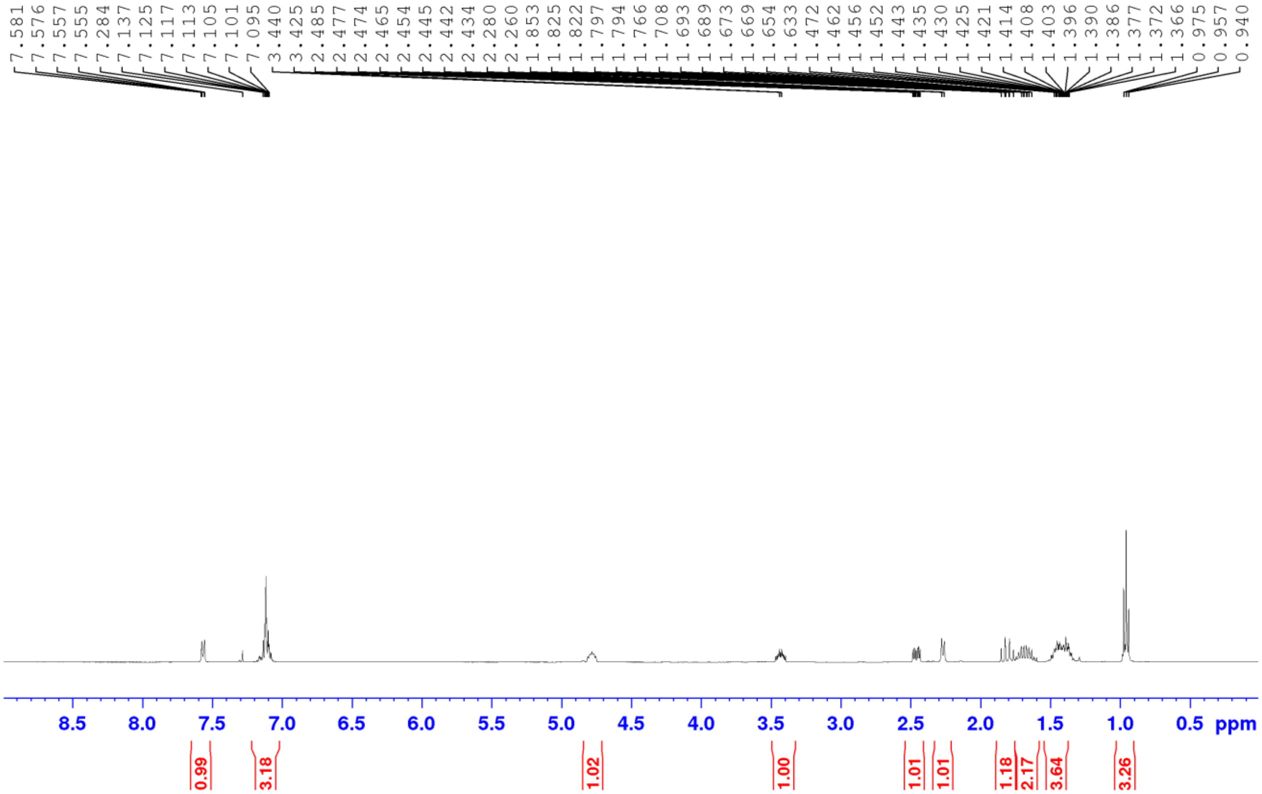

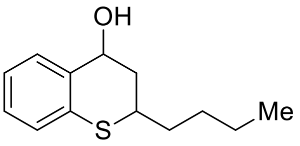

5

2-n-butylthiochroman-4-ol

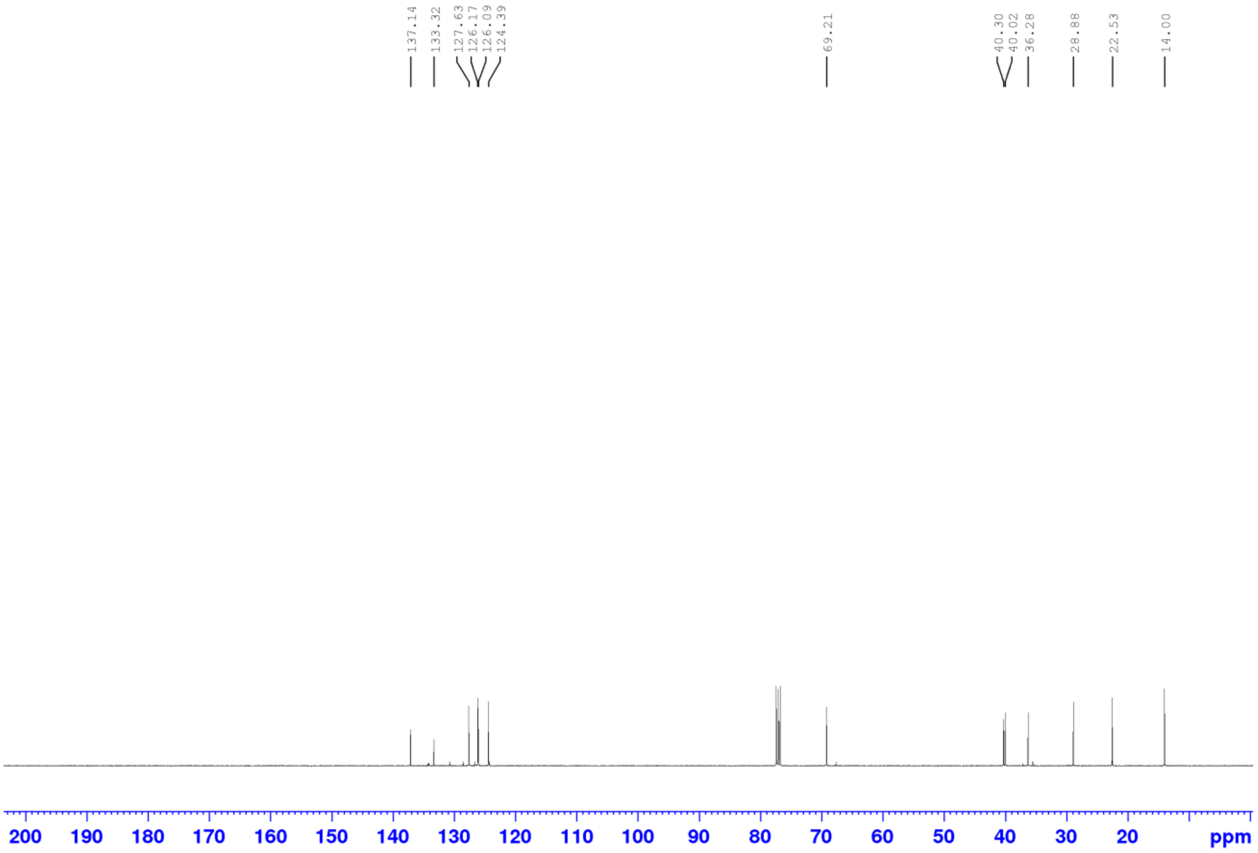

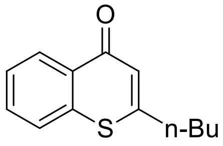
O
S
n-Bu

6

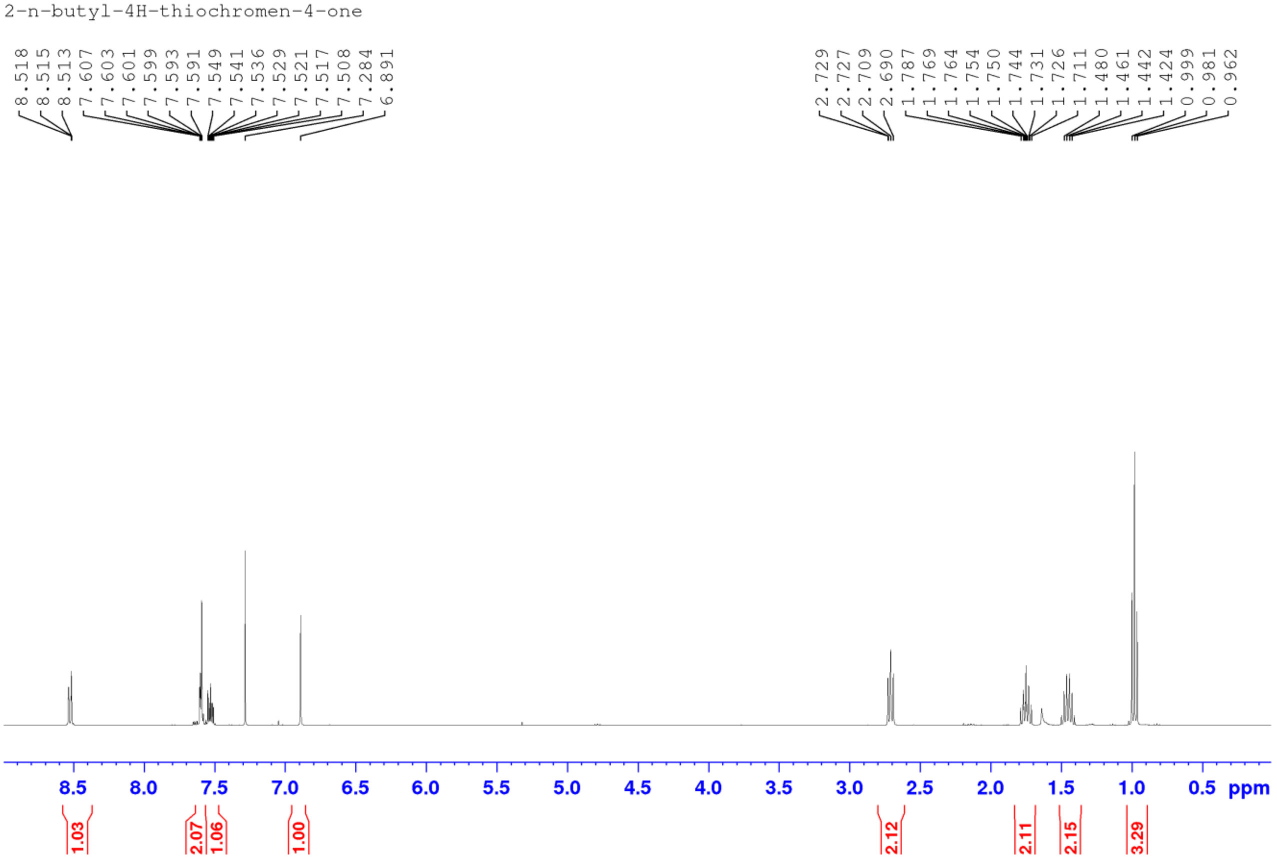
2-n-butyl-4H-thiochromen-4-one
8.518
8.515
8.513
7.607
7.603
7.601
7.599
7.593
7.591
7.549
7.541
7.536
7.529
7.521
7.517
7.508
7.284
6.891
2.729
2.727
2.709
2.690
1.787
1.769
1.764
1.754
1.750
1.744
1.731
1.726
1.711
1.480
1.461
1.442
1.424
0.999
0.981
0.962
8.5
8.0
7.5
7.0
6.5
6.0
5.5
5.0
4.5
4.0
3.5
3.0
2.5
2.0
1.5
1.0
0.5
ppm
1.03
2.07
1.06
1.00
2.12
2.11
2.15
3.29

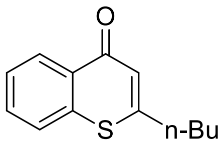
O
S
n-Bu

6

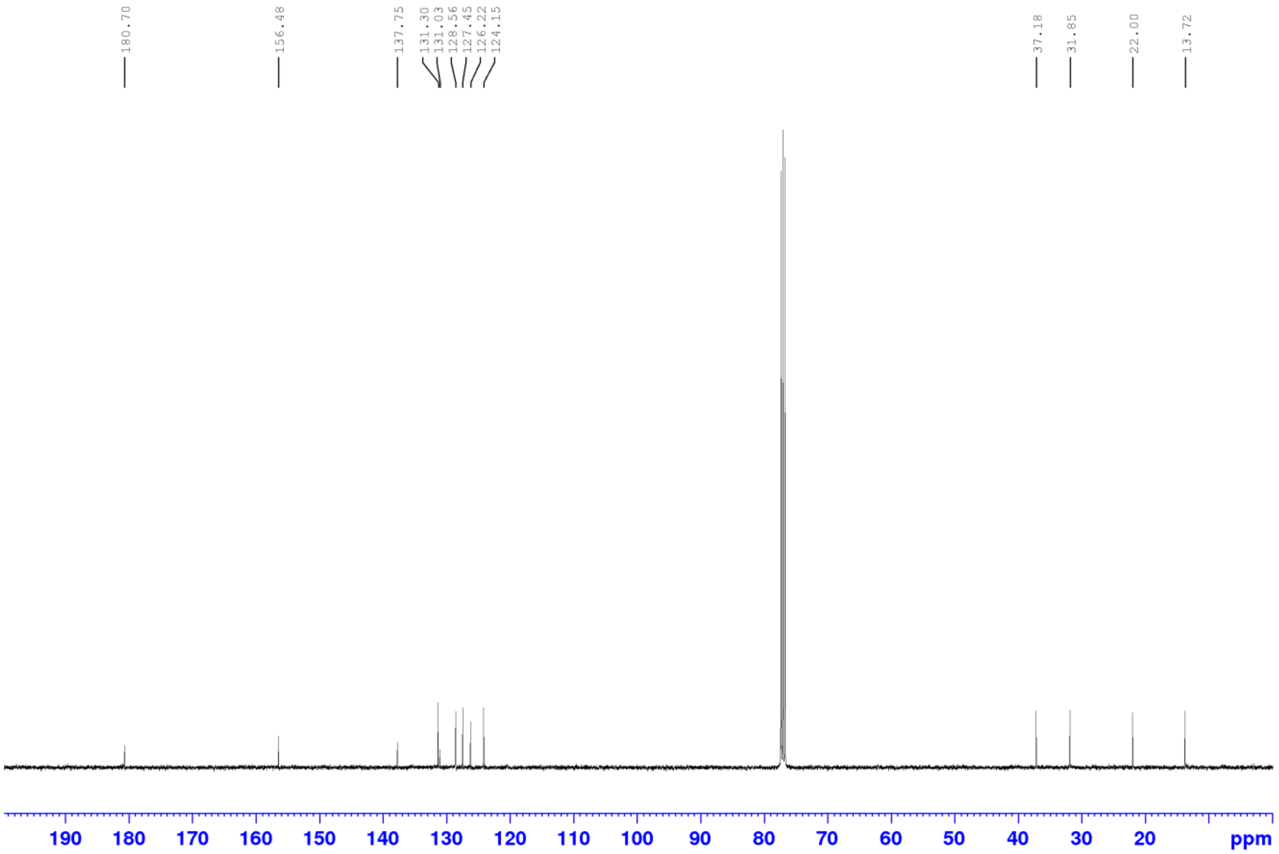
180.70
156.48
137.75
131.30
131.03
128.56
127.45
126.22
124.15
37.18
31.85
22.00
13.72
190 180 170 160 150 140 130 120 110 100 90 80 70 60 50 40 30 20 ppm

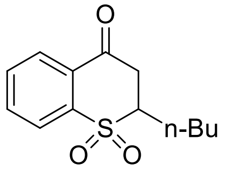

7

2-n-butylthiochromen-4-one 1,1-dioxide

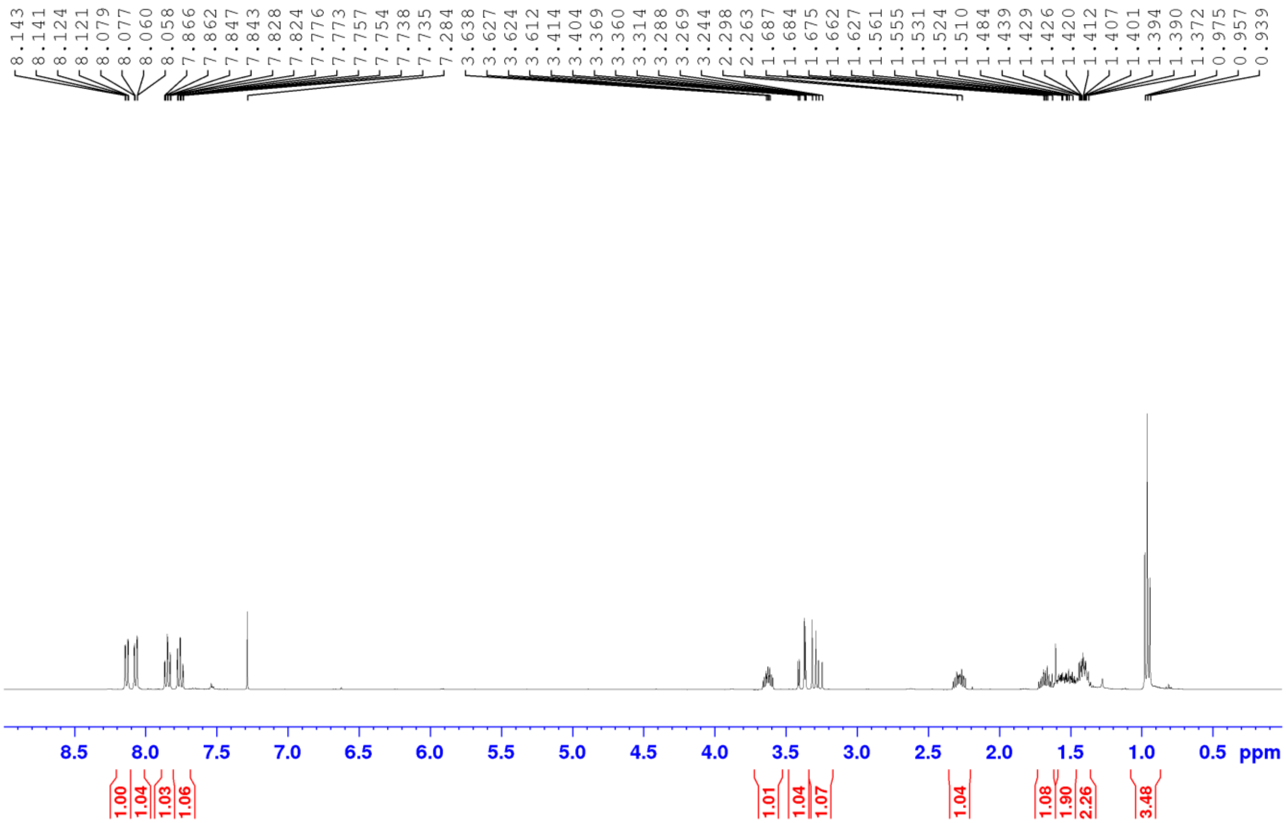

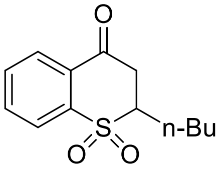
O
n-Bu
S
O
O

7

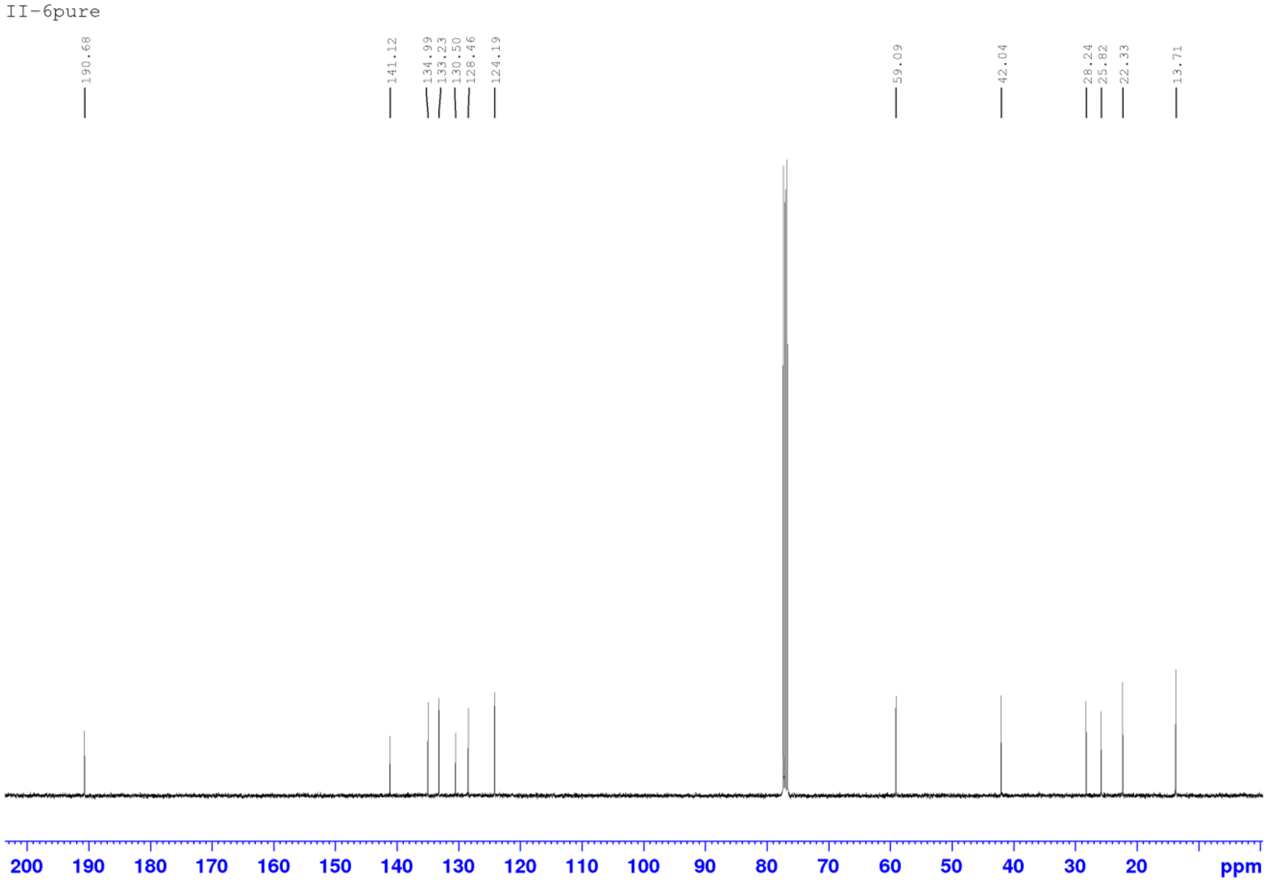
II-6pure
190.68
141.12
134.99
133.23
130.50
128.46
124.19
59.09
42.04
28.24
25.82
22.33
13.71
200
190
180
170
160
150
140
130
120
110
100
90
80
70
60
50
40
30
20
ppm

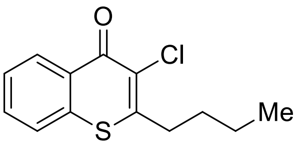

8

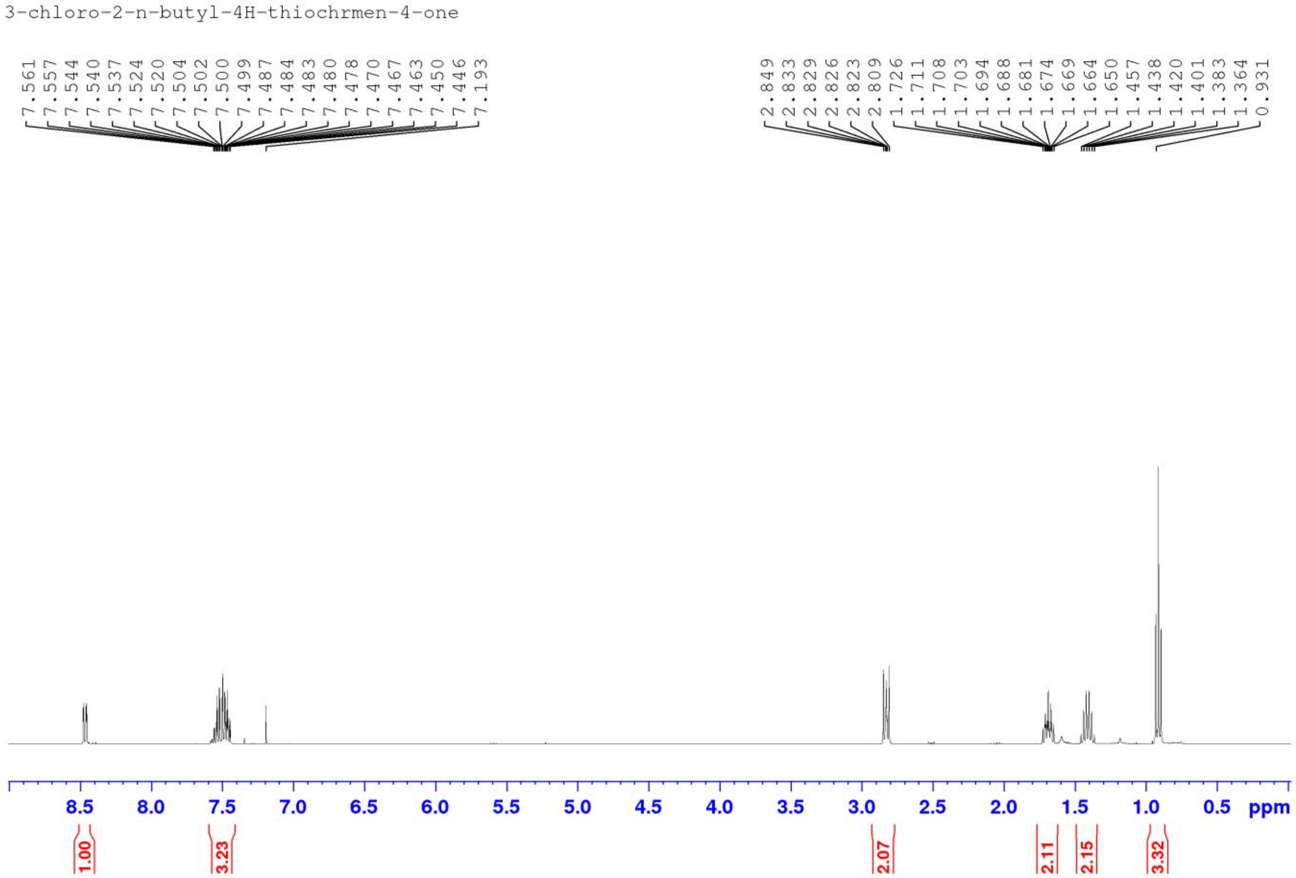

3-chloro-2-n-butyl-4H-thiochrmen-4-one
7.561 7.557 7.544 7.540 7.537 7.524 7.520 7.504 7.502 7.500 7.499 7.487 7.484 7.483 7.480 7.478 7.470 7.467 7.463 7.450 7.446 7.193
2.849 2.833 2.829 2.826 2.823 2.809 1.726 1.711 1.708 1.703 1.694 1.688 1.681 1.674 1.669 1.664 1.650 1.457 1.438 1.420 1.401 1.383 1.364 0.931
8.5 8.0 7.5 7.0 6.5 6.0 5.5 5.0 4.5 4.0 3.5 3.0 2.5 2.0 1.5 1.0 0.5 ppm
1.00 3.23 2.07 2.11 2.15 3.32

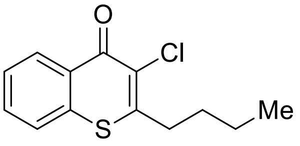
O
Cl
S
Me

8

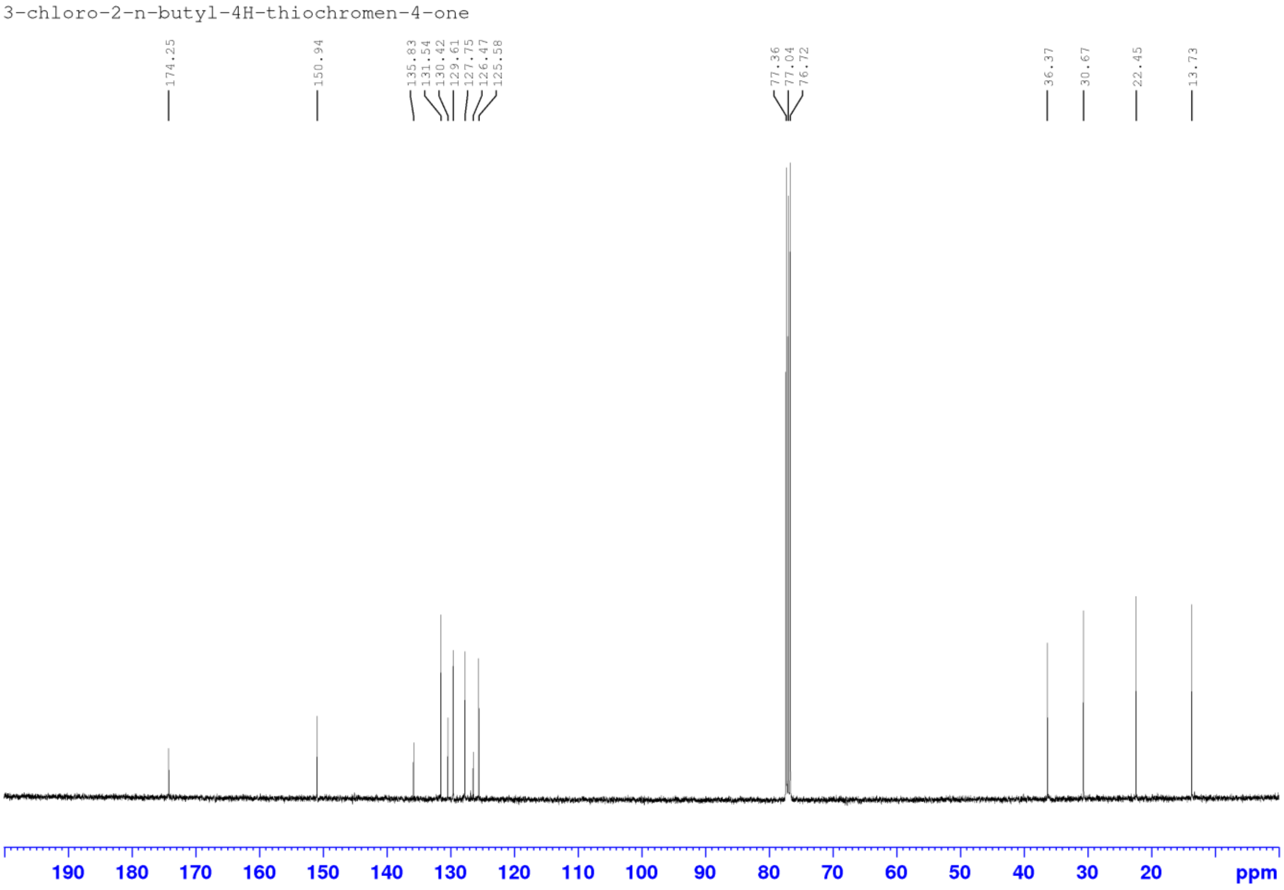
3-chloro-2-n-butyl-4H-thiochromen-4-one
174.25
150.94
135.83
131.54
130.42
129.61
127.75
126.47
125.58
77.36
77.04
76.72
36.37
30.67
22.45
13.73
190
180
170
160
150
140
130
120
110
100
90
80
70
60
50
40
30
20
ppm
